# The Influence of Cathode Degradation Products on the Anode Interface in Lithium-Ion Batteries

**DOI:** 10.1021/acsnano.3c10208

**Published:** 2024-03-20

**Authors:** Zhenyu Zhang, Samia Said, Adam J. Lovett, Rhodri Jervis, Paul R. Shearing, Daniel J. L. Brett, Thomas S. Miller

**Affiliations:** † Electrochemical Innovation Lab, Department of Chemical Engineering, University College London, Torrington Place, London, WC1E 7JE, U.K.; ‡ The Faraday Institution, Quad One, Becquerel Avenue, Harwell Campus, Didcot, OX11 ORA, U.K.; § Renewable Energy Group, Department of Engineering, Faculty of Environment, Science and Economy, University of Exeter, Penryn Campus, Penryn, TR10 9FE, U.K.; ∥ Department of Engineering Science, University of Oxford, Parks Road, Oxford, OX1 3PJ, U.K.

**Keywords:** electrochemical atomic force microscopy, electrochemical quartz crystal microbalance, electrochemical impedance spectroscopy, EC-AFM, NMC, transition metal ions

## Abstract

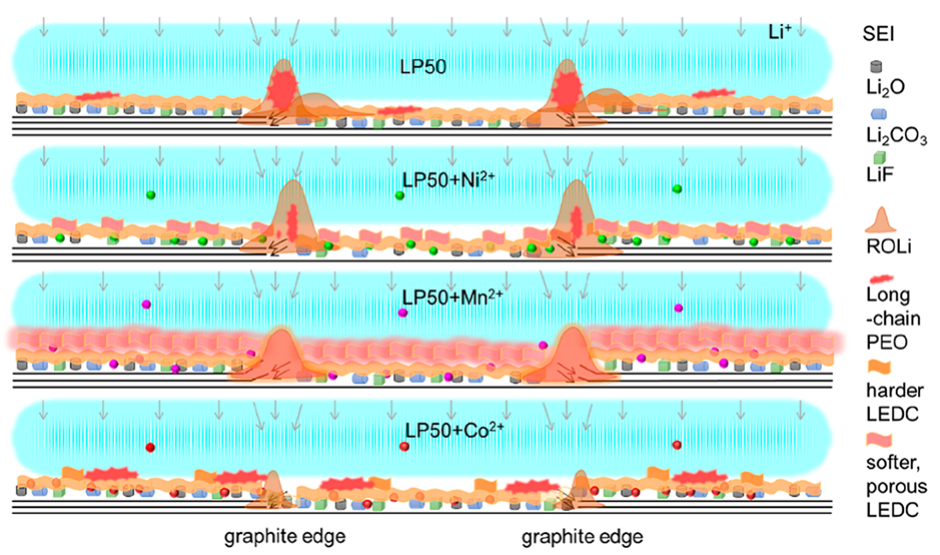

Degradation of cathode materials in lithium-ion batteries results in the presence of transition metal ions in the electrolyte, and these ions are known to play a major role in capacity fade and cell failure. Yet, while it is known that transition metal ions migrate from the metal oxide cathode and deposit on the graphite anode, their specific influence on anode reactions and structures, such as the solid electrolyte interphase (SEI), is still quite poorly understood due to the complexity in studying this interface in operational cells. In this work we combine *operando* electrochemical atomic force microscopy (EC-AFM), electrochemical quartz crystal microbalance (EQCM), and electrochemical impedance spectroscopy (EIS) measurements to probe the influence of a range of transition metal ions on the morphological, mechanical, chemical, and electrical properties of the SEI. By adding representative concentrations of Ni^2+^, Mn^2+^, and Co^2+^ ions into a commercially relevant battery electrolyte, the impacts of each on the formation and stability of the anode interface layer is revealed; all are shown to pose a threat to battery performance and stability. Mn^2+^, in particular, is shown to induce a thick, soft, and unstable SEI layer, which is known to cause severe degradation of batteries, while Co^2+^ and Ni^2+^ significantly impact interfacial conductivity. When transition metal ions are mixed, SEI degradation is amplified, suggesting a synergistic effect on the cell stability. Hence, by uncovering the roles these cathode degradation products play in operational batteries, we have provided a foundation upon which strategies to mitigate or eliminate these degradation products can be developed.

## Introduction

Lithium-ion batteries (LIBs), with graphite anodes and layered NMC (LiNi_*x*_Mn_*y*_Co_1–*x*–*y*_O_2_, *x* ≥ 0.5) cathodes, have been commercialized in electric vehicles because of their high specific capacity and thermal stability.^1^ Nickel-rich NMC (e.g., LiNi_0.8_Mn_0.1_Co_0.1_O_2_, or NMC811) is particularly attractive, as it can achieve a notably high specific capacity (200–220 mAh g^–1^ at a relatively high average discharge voltage of ∼3.8 V vs Li/Li^+^) and may offer lower material costs and environmental impacts compared to its (more Co rich) counterparts.^2,3^ However, despite the promising energy density (∼800 Wh kg^–1^) of NMC811, the development of graphite/Ni-rich NMC cells has been hindered by the increased reactivity of the cathode to the electrolyte at high Ni contents, which negatively impacts cycle lifetimes and cell stability.^4^

In particular, the dissolution of transition metals (TMs) from the NMC cathode and the instability of the solid electrolyte interphase (SEI) at the anode are two of the key phenomena that are known to be responsible for degradation in LIBs. Both chemical and electrochemical processes drive metal loss from NMC cathodes. For example, corrosion can be triggered by electrolyte decomposition to form HF or other acidic species, due to the reaction between LiPF_6_ and trace amounts of water in the electrolyte.^6^ It has also been suggested that organic anions that chelate the metal ions can be formed in operational cells.^7^ Alternatively, electrochemical dissolution has been shown to be exacerbated by aggressive cycling conditions (high temperatures, elevated upper cutoff voltages) with consequences correlated with other cathode degradation mechanisms, such as lattice structure change, rock-salt layer formation, O_2_ and CO_2_ gas evolution, rapid electrolyte decomposition, intra- and intergranular cracks, and capacity attenuation.**(8,9)** The dissolved TM ions are likely to be in the form of lower valence species (e.g., Ni^2+^, Mn^2+^, and Co^2+^) due to their higher solubility in common organic solvents used in LIB electrolytes.^10,11^ Interestingly, it has even been suggested that metal ions can dissolve into the electrolyte from the stainless steel used in cell components.^12^

The presence of TM ions in LIB electrolytes is an important issue, as they have been shown to migrate and deposit on the anode. This “crosstalk” can result in capacity losses in full cells, in particular linked to cell “slippage”, as the TMs can promote side reactions which drive the shifting of the potential profiles of the electrodes, leading to accelerated degradation and capacity fading.^13,14^ One major driver of this slippage is the influence of these ions on the structure and physicochemical properties of the SEI; Mn^2+^ has been suggested to be a particular issue due to a higher tendency for electrocatalysis.^15,16^ Ideally, the SEI structure should be formed solely during the first cycle, be electrically insulating and ionically (Li^+^) conductive, and have good chemical and mechanically stability.^17^ Unfortunately TMs have been shown to affect SEI stability and integrity.^18−21^ For example, X-ray photoelectron spectroscopy (XPS) has been used to demonstrate that Mn ions can easily convert inorganic species in the SEI into Mn-containing compounds such as MnF_2_ through ion-exchange or metathesis reactions, resulting in capacity fade and impedance rises,^22^ while X-ray diffraction (XRD) and Raman spectroscopy analysis have confirmed that dissolved Mn ions can co-intercalate into graphite and lead to structural disordering, inhibiting the intercalation of Li ions.^23^ It has also been suggested that ion-exchange reactions between Ni^2+^ and Li^+^ ions in the SEI can increase SEI resistivity during a long-term cycling, through a combination of time-of-flight secondary ion mass spectroscopy (TOF-SIMS) and electrochemical impedance spectroscopy (EIS) measurements.^24^

Although the chemistry and distribution of TM cathode degradation species on graphite anodes have been studied via various spectroscopies, the impact of the TM dissolution on the morphology, mechanical properties, and stability of the SEI layer remains ambiguous. In our previous work we have demonstrated the ability of *operando* electrochemical atomic force microscopy (EC-AFM) to visualize the SEI formation process at operational battery anodes, observing SEI distribution, evolution, and the impact of electrolyte additives.^25^ Herein, through a combination of *operando* EC-AFM, electrochemical quartz crystal microbalance (EQCM), and EIS measurements, the impact of representative quantities of cathode degradation products on SEI formation and stability is investigated in commercially relevant electrolytes. By deliberately adding TM ions (Ni^2+^, Mn^2+^, and Co^2+^) to the electrolytes of operational LIBs, the mechanisms underlying their participation in the structuring, modification, and destabilization of forming and as-formed SEI layers is revealed. The results gained both highlight that SEI formation is a complex and easily influenced chemical process and offer insights into methods that can be used to develop solutions to stabilize the SEI throughout its operational lifetime.

## Results and Discussion

### Electrochemical Analysis of the TM-Containing Electrolytes

**Figure 1:**
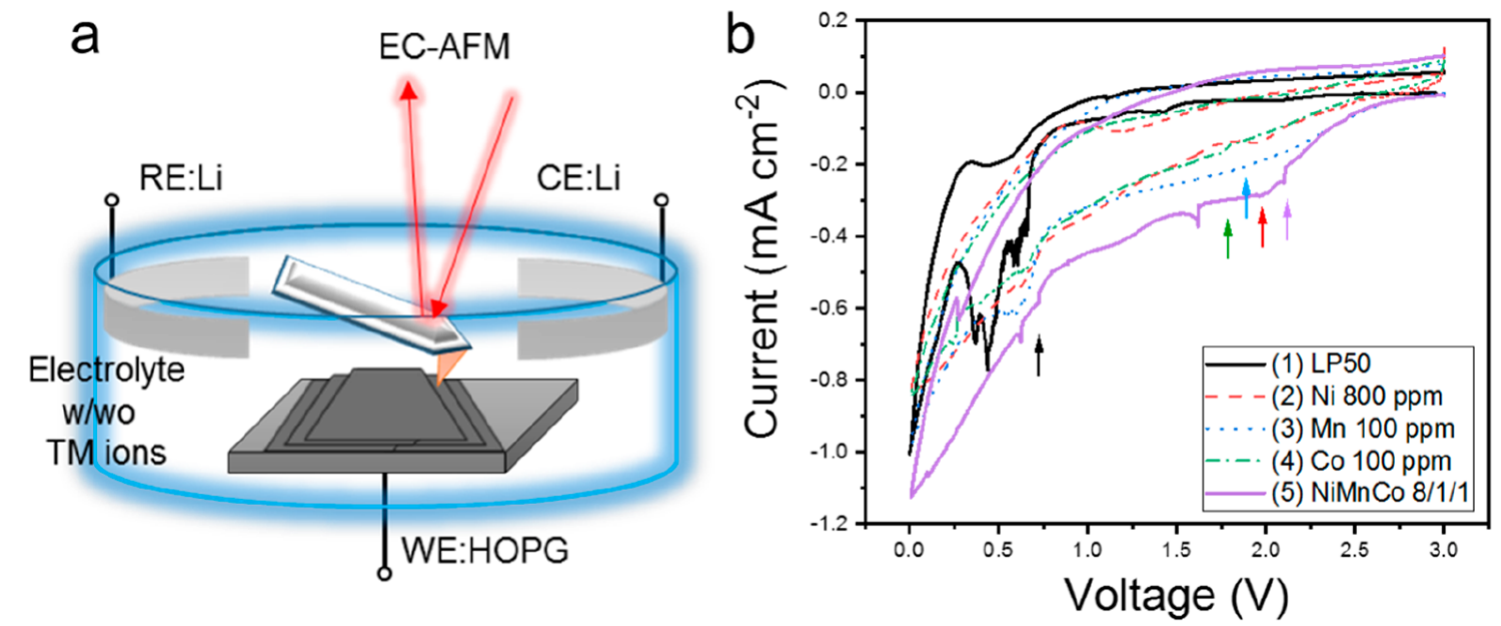
(a) Schematic image of the *operando* EC-AFM cell, showing the working electrode (HOPG) and the reference and counter electrodes (Li metal), highlighting the interaction of the AFM probe with the electrode surface. (b) As recorded CV curves during the *operando* EC-AFM experiments, conducted between 3.0 and 0.01 V at a scan rate of 0.5 mV s^–1^, within the 5 different electrolytes (1) LP50, (2) LP50 + 800 ppm of Ni^2+^, (3) LP50 + 100 ppm Mn^2+^, (4) LP50 + 100 ppm of Co^2+^, (5) LP50 Ni^2+^/Mn^2+^/Co^2+^ 800/100/100 ppm.

To assess the impact of TM ions on the SEI of LIBs, a graphite anode (highly oriented pyrolytic graphite, HOPG) was cycled vs Li in electrolytes based on a commercial LP50 electrolyte containing 1 M LiPF_6_ in ethylene carbonate/ethyl methyl carbonate (EC/EMC); pure LP50 is referred to as electrolyte 1. The LP50 was in turn dosed with Ni^2+^ 800 ppm (electrolyte 2), Mn^2+^ 100 ppm (electrolyte 3), Co^2+^ 100 ppm (electrolyte 4), or a mix of Ni:Mn:Co 800:100:100 ppm (electrolyte 5), i.e., stoichiometric to the NMC811 cathode composition. For comparison, an electrolyte containing 100 ppm of Ni^2+^ was also tested. The configuration of the EC-AFM cell is shown in Figure 1a.

Cyclic voltammetry (CV) curves collected in the AFM electrochemical cell containing each of the five electrolytes, conducted between 3.0 and 0.01 V at a scan rate of 0.5 mV s^–1^, are shown in Figure 1b. Note: All voltages throughout this article are quoted vs Li/Li^+^. In the cathodic scan of the pure LP50 electrolyte, i.e., electrolyte 1, the current–voltage response is largely as expected; little current is measured above ∼0.75 V, and below this point a significant reductive current is measured corresponding to the decomposition of EC and formation of the SEI layer on the graphite surface.^26^ At lower potentials, Li intercalation also contributes to the Faradaic current measured. However, it can clearly be seen that the addition of any of the individual TM ions studied induces a significant electrochemical change. With TM ions in the electrolytes, the current density is significantly increased from the start of the cathodic scan, and for electrolytes 2 and 4, i.e., in electrolytes containing Ni and Co ions, small reduction peaks can be observed at ∼2.0 and 1.75 V, respectively. Comparatively, the reduction of Mn^2+^ (electrolyte 3) induces an even higher current density with a broad contribution between 2.5 and 1.0 V. A previous study by Jung et al. linked peaks in this region to the reduction of TM ions, specifically 1.27, 2.22, and 2.52 V for reduction of Mn^2+^, Ni^2+^, and Co^2+^ ions;^27^ however, here peaks are significantly smaller in magnitude as the TM ion concentration (0.8, 0.1, and 0.1 mM of Ni(TFMS)_2_, Mn(TFMS)_2_, and Co(TFMS)_2_, respectively, in this work vs 60 mM of Ni(TFSI)_2_, Mn(TFSI)_2_, and Co(TFSI)_2_ in the reference) is between 75 and 600 times lower to ensure quantities are representative of the concentrations of cathode degradation products expected. Anions of TFMS^–^ are not expected to play any significant role, due to their stability and low concentration compared to PF_6_^–^ in the electrolyte. In electrolyte 5, with a mixed TM ion composition, the CV response appears to be largely cumulative based on the CVs from the individual ions, with a possible slight lowering of the TM ion reduction overpotential.

Importantly, the same overall processes can be seen to occur at similar potentials in both the EC-AFM cell and coin cell anodes (e.g., onset potentials for SEI formation) (Figure S1, Supporting Information), even though there are clear differences between the CV curves. However, these differences can be explained by the differences in the specific properties of carbon materials and cells used (HOPG electrode in an EC-AFM cell, tested at a scan rate of 0.5 mV s^–1^, vs graphite composite electrode in the coin cells at a scan rate of 0.05 mV s^–1^; graphite with a particle size of ∼10 μm, mixed with PVDF and carbon black in a ratio of 90/5/5, loading 13 mg cm^–2^). In the CV conducted on HOPG, the electrochemistry is dominated by the formation of an SEI, as HOPG has very few exposed edge sites to allow intercalation. In the coin cell electrode, the graphite has an abundance of step edges, so the intercalation process dominates the observed response. Importantly, however, as can be seen most clearly in the pure LP50 electrolyte, key electrode phenomena occur at each electrode at similar potentials vs Li/Li^+^, which is vital for analyzing the EC-AFM response. For example, SEI formation can be seen to initiate at ∼0.75 V. It should be noted that there is an overall increase in capacitive contributions in the EC-AFM cell compared to the coin cell, which may be partially attributed to the higher scan rates of the CV tests (10 times higher) that increases the capacitive background. However, the increase in current density is primarily due to the initiation of the accumulation of species (TM or SEI) on the surface, rather than capacitance. Hence, it is essential that advanced surface characterization tools are utilized to elucidate the impact of cathode degradation products at the anode.

### *Operando* EC-AFM of SEI Formation

**Figure 2:**
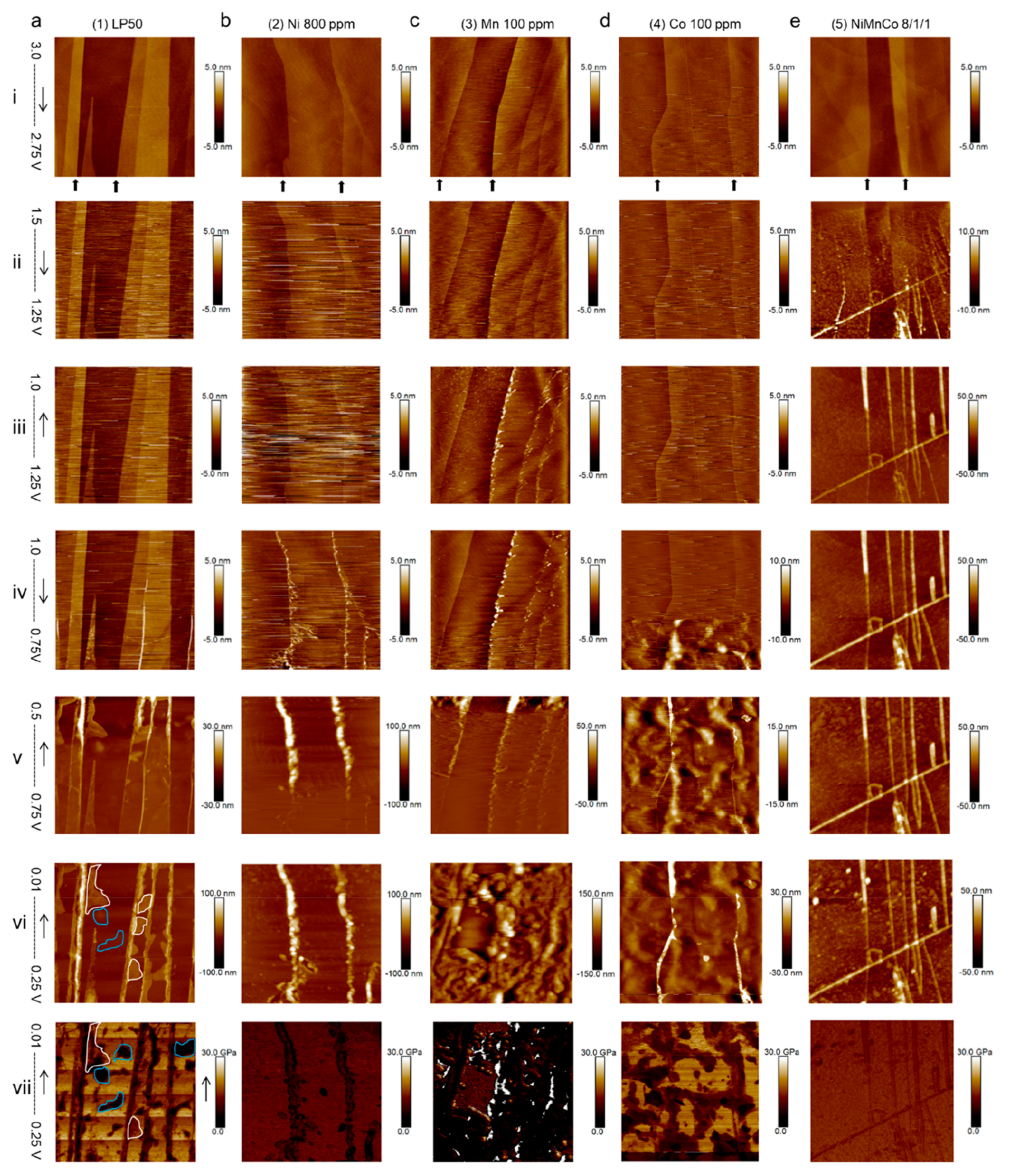
EC-AFM images (10 × 10 μm scan area) of (row i) pristine HOPG, SEI formation in the voltage range of 1.5–1.25 V (row ii), 1.25–1.0 V (row iii), 1.0–0.75 V (row iv), 0.75–0.5 V (row v), and corresponding morphology (row vi) and modulus (row vii) images between 0.25–0.01 V. Columns (a)–(e) present SEI formation in the five electrolytes a. (1) LP50, b. (2) LP50 + 800 ppm Ni^2+^, c. (3) LP50 + 100 ppm Mn^2+^, d. (4) LP50 + 100 ppm Co^2+^, e. (5) LP50 Ni^2+^/Mn^2+^/Co^2+^ 800/100/100 ppm. The scan direction (left) and height scale bars (right) are shown in the images.

Figure 2 shows *operando* EC-AFM images collected in electrolytes 1 to 5. While row (i) displays images of each of the freshly cleaved areas of HOPG studied (while being cycled between 3.0 and 2.75 V, a range where no significant current was passed in any electrolyte), rows (ii)–(v) show the morphology change during SEI formation (1.5–0.5 V). Correspondingly row (vi) and row (vii) show morphological changes and maps of the surface modulus (in GPa), respectively, in the voltage range of 0.25–0.01 V. Full data sets that include the images shown in Figure 2 (morphology and modulus mapping) are displayed in Figures S2–S8. The freshly cleaved HOPG had a very clean and smooth surface after it was submerged into the electrolytes, with observable graphite step heights of ∼1–2 nm (3–6 carbon layers). The initial modulus of the HOPG surface was calibrated to between 18 and 20 GPa.^28^

In the pure LP50 electrolyte (1), the morphology change above ∼0.9 V was subtle, except for a little disturbance of the scanning probe, which caused the horizontal lines in the images. This phenomenon might be attributed to the complicated local environment of the scanning probe in the liquid, in which the composition is changing due to capacitive and redox processes, as well as electrolyte composition changes; there are numerous factors that could cause artifacts.^29^ As at the beginning of scanning (3.0 V) the image is clear without any artifacts and the image is similarly clear at low potentials, and as this phenomenon occurs repeatably in different experiments, we believe changes in the liquid electrolyte density or viscosity could be a reasonable explanation. Importantly, there is significant evidence in the literature that AFM and related techniques can be used to map changes in the electrolyte structure (e.g., double layer),^30,31^ and hence it is known that electrolyte restructuring can impact the behavior of the AFM tip. It should be noted that the height and modulus mapping data were obtained simultaneously hence the horizontal artifact lines for morphology and modulus are synchronized. The corresponding modulus images (Figure S2) show modulus values increased as the potential dropped, which may be caused by the generation of initial polycrystalline or amorphous inorganic SEI species such as LiF or Li_2_CO_3_ above 1.0 V.^32^ Major changes in the morphology and modulus did, however, occur below ∼0.9 V (Figure 2a, iv), where the measured height at step edges began to increase, while the edge modulus drops. This can be assigned to the increasing reduction of EC, via the single-electron pathway, to generate lithium ethylene dicarbonate (LEDC).^33^ As the potential dropped further, the accumulation of SEI at the edge continued (up to 125 nm at 0.01 V) and the step edge modulus similarly fell further (∼3 GPa at 0.01 V), as shown in Figure 2a, vi and vii. The majority of the basal plane areas exhibited a high modulus of ∼16 GPa throughout the cycling, while the SEI thickness remained low (∼10 nm, as calculated by indentation depth measurements (Figure S3d and g). This may indicate a higher content of amorphous inorganic species in the SEI (such as LiF and Li_2_CO_3_, 13.3–45.5 GPa).^32^ However, as the potential dropped toward 0.01 V, distinct low-modulus areas did appear on the basal planes, both in regions linked to step edges (highlighted using white lines in Figure 2a, vi and vii; non-highlighted images are shown in Figure S2) and those apparently unlinked to step edges (marked in blue in Figure 2a, vi and vii). Considering the dynamic nature of the SEI, the contrast and resolution of the morphology and mechanical images can be impacted due to enhanced ion flux, especially for the observed undulations around step edges.

As shown in Figure S3, the majority of step-edge-linked low-modulus areas (encircled in white, Figure 2a, vi and vii) on the electrode cycled to 0.01 V in electrolyte 1 had heights of ∼30 nm and modulus values close to 10–12 GPa, while most of the non-step-edge-linked areas (encircled in blue, Figure 2a, vi and vii) were less thick (∼21 nm) and had a lower modulus (∼5 GPa). Similar differences can be seen in the relative (tip–surface) adhesion values of these two areas (Figure S3c), with the areas marked in blue (non-edge-linked) showing stronger adhesion values, while the white marked areas show less adhesion to the tip. Together, these data may suggest a different composition for these two feature types. It has previously been shown that the composition of SEI is location dependent,^32^ and while the modulus values are too low to suggest a simple inorganic/polymeric SEI separation of the two areas, the differences measured may indicate different stoichiometries of common SEI components or even the presence of different polymeric species (e.g., LEDC 8.4–18.9 GPa, PEO 0.9–2.5 GPa).^32^ The behavior of these two regions upon charging (Figure S2) may also support the conclusion that they are different; at the as-formed edge SEI destabilizes and detaches, while the basal plane accumulations appear more stable.

It is worth noting that compared to our previous work,^25^ which used EC/EMC 3/7 (v/v, LP57), a higher EC content electrolyte (EC/EMC 1/1 (v/v, LP50)) is used here. It has been demonstrated that the relative composition of EC reduction products is dependent on its concentration; Li_2_CO_3_ is more likely to form at a low EC concentration, while LEDC is more likely to form at a high EC concentration.^33^ This agrees well with the observation here that the SEI formed is somewhat less particulate in nature and less stable (Figure S2).

In electrolyte 2, which contained Ni^2+^ ions, the morphological change above 1.0 V was similar to that in electrolyte 1 (Figure 2b, iii), but below this voltage, small particles with a slightly lower modulus than graphite formed at the step edges. At ∼0.65 V significant thickening (∼100 nm) and softening (modulus drops to ∼5 GPa) of the SEI could be observed, leaving much more significant edge SEI deposits than that found in pure LP50. After lithiation, the edge SEI in electrolyte 2 was taller and wider (Figure 2b, vi) than that of electrolyte 1, while large particles (width of 100–500 nm and height of 50–100 nm) of material (likely SEI) could be observed on the basal plane. Here the overall modulus of the whole surface was lowered as the anode approached 0.01 V (edge modulus of ∼5 GPa and basal of ∼11 GPa, as shown in Figure 2b, vii), indicating a more significant growth and uniformly distributed soft polymeric materials, such as LEDC and PEO, than those observed in electrolyte 1. The as-formed edge SEI was unstable during charging from 0.01 V, which would subsequently lead to additional SEI formation and SEI thickening on further cycles (Figure S4), lowering Coulombic efficiency.^34^

For a fair comparison with electrolytes 3 and 4, the SEI formation in LP50 containing 100 ppm of Ni-TFMS was also tested (Figure S5). With the lower concentration of Ni^2+^, the morphology and mechanical properties of the SEI layer were consistent with the 800 ppm data, including the observation of thickened edge SEI and the continued presence of particles on the basal areas, but to a lower magnitude, meaning the data also had similarities to those collected in electrolyte 1. At 0.01 V, clear particles with diameters of 100–200 nm could be observed (zoom-in images, Figure S5). Note: A zoomed-out image at 0.01 V (Figure S5) reveals that the influence of the AFM probe on the morphology and modulus images was negligible.

The data in Figure 2c demonstrate that Mn^2+^ ions have a major influence on the structure and properties for the SEI in LIBs; in electrolyte 3 the SEI formation process was significantly different from that in electrolyte 1 or 2. Below 1.25 V, a much higher onset potential, particles could be seen to precipitate at the HOPG edges, leading to a broken and softened edge structure (Figure 2c, iii and iv). Mn^2+^ has previously been observed to co-intercalate into graphite and damage the layered structure.^23^ A major structural change was then observed below 0.55 V, where the edge SEI height suddenly increased from a height of ∼10 to over 100 nm, which was quickly accompanied by the accumulation of thick and soft SEI across the majority of the graphite surface (Figure 2c, vi). In the same voltage range, the modulus across almost the whole surface, excluding one small patch, could be seen to sharply drop to ∼1 GPa (Figure 2c, vii). Note: Due to the extreme softening of the surface, the spring constant of the tip was a nonideal match, leading to the white mismeasurement/error zones in Figure 2c, vii. These areas do not represent surface hardening. It is widely accepted that the decomposition of the organic solvent and generation of the SEI layer are promoted by manganese species in the electrolyte.^15^ The observations here agree well with this conclusion.

Co^2+^ is demonstrated to impact SEI structures in a different way from Ni^2+^ and Mn^2+^ in Figure 2d, with particular impact on basal plane areas. In electrolyte 4, interphasial changes began at ∼0.85 V, where large areas of low-height (<15 nm) and low-modulus (5–7 GPa) materials appeared first at the basal plane (Figure 2d, iv). Different from other cases, the edge SEI then began to evolve at lower potentials. Interestingly, however, little change was observed in the basal SEI after it had formed (<30 nm thick), while the height and width of the edge SEI continuously grew, up to 50 nm, as the voltage dropped toward 0.01 V, as shown in Figure 2d, vi.

**Figure 3:**
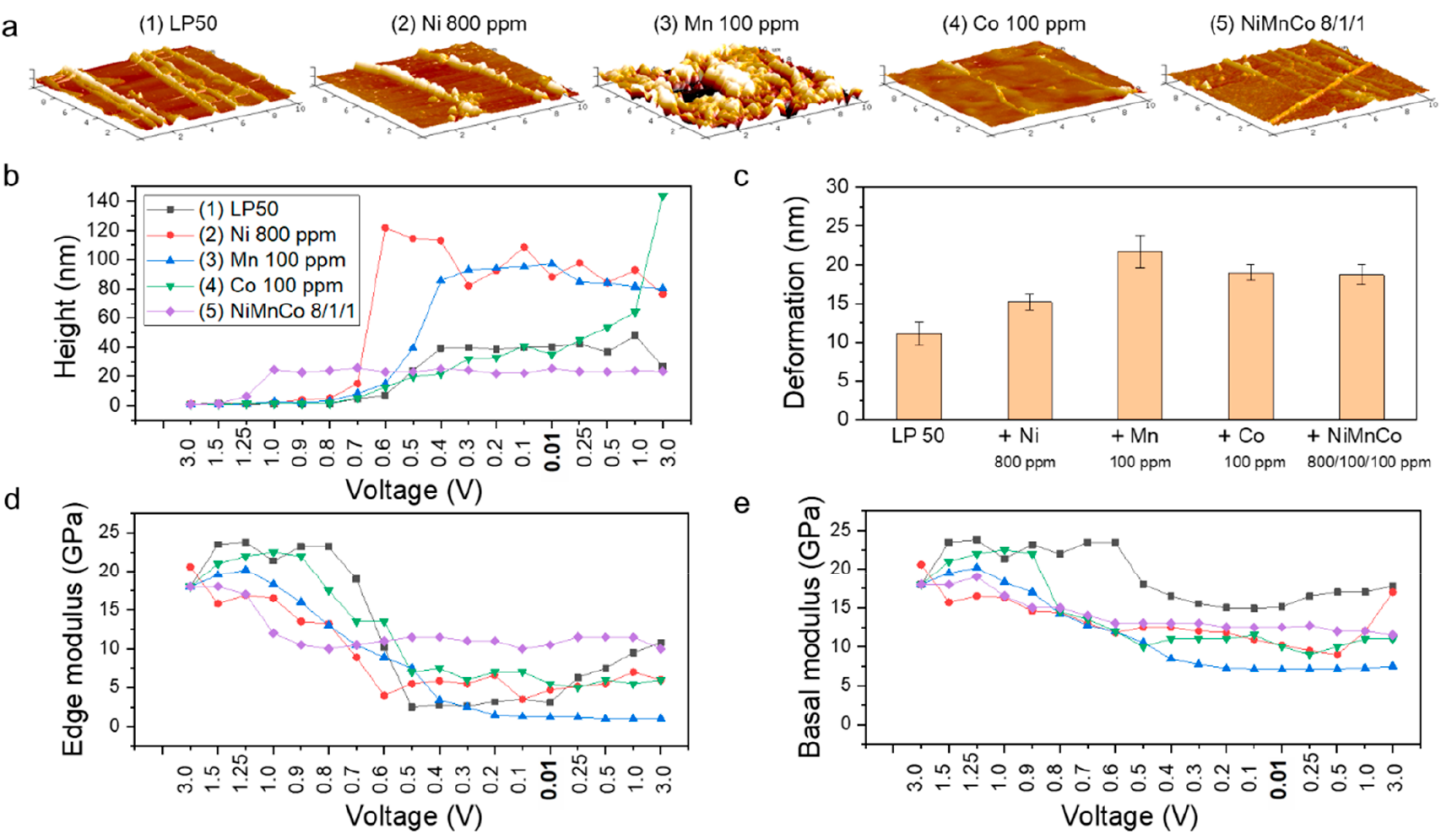
(a) 3D representations of the images in Figure 2, vi, with the same height scale of 100 nm, allowing the comparison of the SEI layer thickness in different electrolytes. (b) A plot of the evolution of edge SEI height during the first lithiation and delithiation in the five electrolytes. (c) Bar charts of the deformation (obtained from the average indentation depth of the AFM probe, implying the thickness) of basal SEI at 0.01 V. Modulus evolution at (d) edge and (e) basal areas on the HOPG during electrochemical discharge and charge processes, in the five electrolytes.

Finally, the impact of a stoichiometric mix (relative to NMC 811) of TM ions was assessed (electrolyte 5). Interestingly here, the phenomena observed were clearly not a simple accumulation of all of those occurring in the single TM ion examples, suggesting a complex interplay of influences exerted by the TM ions. First, the onset potential for deposition of species at the graphite surface was significantly elevated (to ∼1.5 V) in the mixed TM ion electrolyte, consistent with the CV data (Figure 1b). In Figure 2e, ii, material can be seen to have accumulated along all step edges and to a lesser degree as particles across the basal planes. As the potential dropped toward 0.75 V (Figure 2e, iv), the SEI continued to accumulate, primarily at step edges, consistent with the behavior in LP50 (electrolyte 1) and Ni^2+^ (electrolyte 2). Upon further lowering of the potential closer to 0.5 V (Figure 2e, v), basal plane roughening and the accumulation of particles could be seen, more consistent with the behavior in electrolytes 2 and 3, containing Ni^2+^ and Mn^2+^. As the voltage approached 0.01 V (Figure 2e, vi and vii), the SEI appeared to stabilize, and little additional change was noted; the final SEI structure consists of step-edge-oriented accumulations and rough and particulate basal plane layers. The average modulus value measured at the basal area in electrolyte 5 was higher than those for electrolytes 2 and 3, but lower than in electrolytes 1 and 4. The SEI accumulated at step edges also appears less soft, possibly indicating a greater concentration of inorganic species. However, while these are often considered to be features of a quality SEI (dense, compact), the SEI formed was found to be unstable upon charge, resulting in the exposure of fresh graphite (Figure S8). 3D images in Figure 3a of the formed SEI layers (2D images in Figure 2, vi) highlight the significant differences in interface structure in the five electrolyte systems.

To further analyze the morphology and mechanical properties of the evolving interphasial layers, the average heights of two edges (initially 1–2 nm thick, indicated by black arrows marked in row (i) of Figure 2) at different voltages were recorded (Table S1) and then normalized (see Methods) before being plotted in Figure 3b. The LP50 data (electrolyte 1) are consistent with that measured previously for HOPG electrodes, indicating a significant thickening of the SEI at step edges below ∼0.7 V.^25^ However as seen in the images in Figure 2, the TM ions appear to impact SEI growth differently. All TM ions appear to induce SEI formation at slightly higher voltages than the pure LP50, while electrolytes with Ni^2+^ (2) and Mn^2+^ (3) induce more SEI at the graphite edge, while electrolytes containing Co^2+^ (4) appear to have little major impact on edge SEI thickness, as does the mixed TM electrolyte (5). However, all of the TM ions in the electrolytes do appear to detrimentally induce thickening of the basal plane SEI (Figure 3c), determined from deformation measurements of the films at 0.01 V (Figure S9). Note that the deformation value is not equal to the absolute thickness (it is usually smaller), but it is helpful to compare the basal SEI between different samples.

Importantly, the results in Figure 3a–c agree with previous studies that indicate the presence of Mn^2+^ in the electrolyte results in a thick SEI layer, not only at graphite edges but across the whole surface. The film measured was consistently close to twice as thick at both edges and basal planes, when compared to the LP50 electrolyte (1). These observations verify the previous reported findings that Mn^2+^ accelerates the SEI growth due to a higher catalytic reactivity of Mn species, resulting in a larger SEI thickness, higher electrode resistance, and capacity fade.^15,20,22,35^ According to a previous report, a Mn^2+^ derived SEI exhibits an unstable porous structure and undergoes severe morphological changes during the electrochemical process.^36^

Figure 3d and e show the average Young’s modulus of the SEI formed at HOPG edges and basal planes as a function of potential (data given in Table S2). Again, the TM ions have a significant but varied impact on the SEI structure. At the graphite edge, the modulus values drop immediately upon formation of an SEI during lithiation for all electrolytes, indicating that the layer formed is detrimentally thick and soft (modulus as low as 1–5 GPa). Surprisingly, with the mixed TM ions in the electrolyte 5, the modulus remains the highest of all the electrolytes throughout the lithiation process (∼10 GPa). Consistent with previous work in the electrolyte LP57,^25^ the SEI formed at the basal planes was generally thinner and harder than that at the step edges (Figure 3e). Electrolyte 1 induced the highest basal modulus, suggesting that the TM ion containing electrolytes promote the generation of a consistently soft, polymer containing SEI, which can lead to inventory losses and cell instability. While the impact of Mn^2+^ has been noted in the literature, the data presented show that Co^2+^ species can in fact induce a basal SEI that is almost as soft and thick, although less covering.

Interestingly, during the anode delithiation process (0.01 to 3.0 V), the LP50 electrolyte (1) appears to demonstrate the most significant edge SEI destabilization behavior, although it should be noted that this is from a baseline of a relatively thick and particularly soft SEI at the graphite edge (Figure S2). It could be suggested, therefore, that the presence of some TM ions may enhance structural stability at the SEI, although their other negative impacts (e.g., slippage) will likely negate any benefit. Ni^2+^ containing electrolytes, however, appear to offer unstable SEI at both the edge and basal planes; the basal plane modulus in particular increases significantly after being charged back to 3.0 V due to SEI detaching from the surface, exposing a fresh HOPG surface.

The above results demonstrate that TM ions significantly influence the structure and properties of the SEI layer during the first lithiation of the graphite anode. However, TM ions are unlikely to be present in significant quantities during the formation of SEI in the first discharge–charge cycle in real-world batteries. Therefore, the effects of TM ions on SEI degradation after SEI formation were conducted here by *ex situ* AFM characterization. First an SEI layer formed in pure LP50 was imaged (first CV cycle in LP50 (1)), then the impact of cycling this SEI layer in a TM ion containing electrolyte was assessed (second CV cycle in electrolyte 5, NiMnCo, 8/1/1). As seen from Figure S10a, after the first cycle in electrolyte 1, LP50, the basal SEI layer observed was composed of a coating of material across the surface with a roughness of ∼10–20 nm, with slightly larger accumulations (∼20–40 nm) at the edge planes. The lower edge SEI height compared to that observed in the *in situ* experiments may be in part due to the fact that the electrode was rinsed to remove salts or due to the fact that this test was performed in a cell with a lower quantity of electrolyte with the anode in contact with a separator. Nonetheless, while it is lower in magnitude, the response is consistent. Figure S10b shows how the SEI morphology developed in the presence of the TM ions (i.e., after the second CV cycle). Clear changes can be observed, primarily an overall thickening of the interphasial layer, particularly at the step edges. The densification of the step edge SEI and roughening of the basal plane are consistent with the operando EC-AFM findings. These results therefore confirm that TM ions in the electrolyte promote the growth of the SEI layer, at both basal and edge planes, even after the SEI is established, as would be the case in commercial LIBs.

### EQCM Study of the Impact of TM Ions

**Figure 4:**
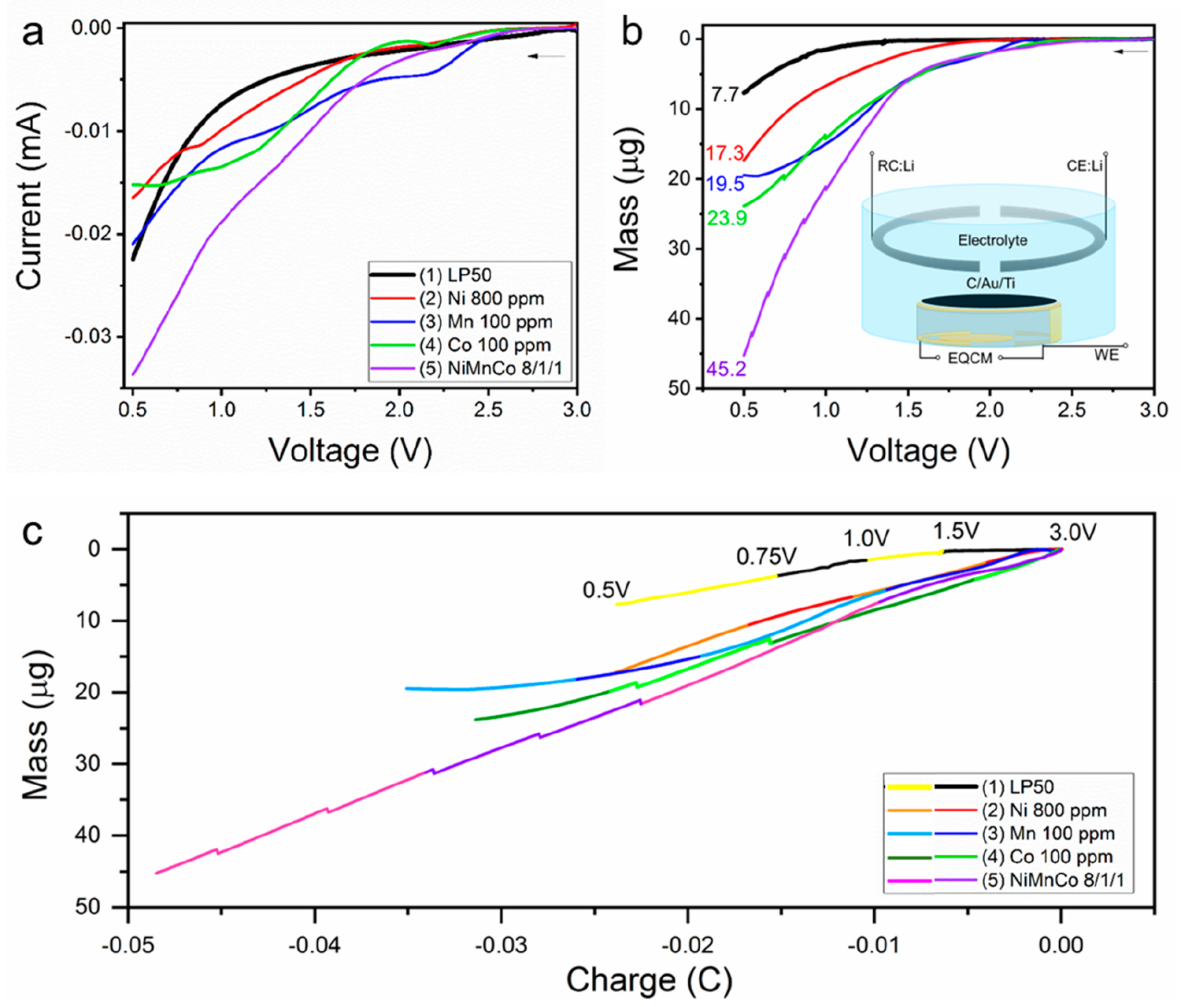
*Operando* EQCM results of the SEI formation on the carbon electrode in different electrolytes during the first discharge. (a) Current–voltage curves obtained from LSV discharge from 3.0 to 0.5 V at a scan rate of 0.5 mV s^–1^. (b) Mass–voltage curves. (c) Mass–charge curves for the EQCM cells with the five different electrolytes during the first discharge process. The mass–charge curves are plotted across voltage ranges of 3.0–1.5, 1.5–1.0, 1.0–0.75, and 0.75–0.5 V for calculation of the corresponding slope and MPE values in each stage.

To help explain the differences in the interphasial phenomena observed by EC-AFM in the five electrolytes, EQCM measurements were performed (see the Methods). EQCM is an advanced tool for monitoring capacitive and Faradaic charge transfer processes at electrode surfaces;^37^ EQCM is particularly useful in the study of interphasial processes in batteries.^38,39^ Here, carbon-coated quartz EQCM sensors were used as working electrodes and cycled in electrolytes 1–5 to help uncover the differences in the SEI formation process in electrolytes with/without added TM ions, as shown in the inset of Figure 4b.

The carbon-coated crystals were first characterized by Raman spectroscopy and AFM (Figure S11), with the Raman spectrum showing a broad peak centered close to 1500 cm^–1^, as expected for carbonaceous materials, which could be fitted with the D (1360 cm^–1^) and G (1560 cm^–1^) components typical of graphitic materials.^40^ AFM showed that the carbon layer was composed of carbon particles with a diameter of ∼100 nm. Although the carbon phase was somewhat different from the HOPG used in the EC-AFM experiments, the SEI formation processes on the surface of the crystal were nonetheless expected to be very similar.^41^ To avoid any alloying reactions between Li^+^ and the Au layer supporting the carbon, the linear sweep voltammetry (LSV) scan was conducted from 3.0 to 0.5 V. Importantly this allowed the study of SEI formation, which was established above this potential, and hence the impact of the TM ions was resolvable.^42,43^

Figure 4a shows the first discharge LSV curves, and Figure 4b shows corresponding mass accumulation curves for the SEI formation process on the carbon-coated EQCM electrodes within the five electrolytes. In electrolyte 1 the discharge shows an onset potential (∼1.0 V) for SEI formation similar to that observed in the EC-AFM cell, below which the current density increased rapidly. Correspondingly, the mass buildup of the SEI layer increased after ∼1.0 V, eventually resulting in an overall SEI mass of ∼7.7 μg at 0.5 V. For electrolytes 2–5, small current peaks above 2.0 V correspond to the reduction of Ni^2+^, Mn^2+^, and Co^2+^ ions, before displaying higher reduction currents between 2.0 and 0.75 V, indicating the catalytic effect of TM ions for electrolyte decomposition and SEI formation at a higher potential, consistent with AFM observations (Figure 2). The higher current led to faster accumulation of SEI mass on the electrode surface: 17.3, 19.5, and 23.9 μg at 0.5 V for the electrolytes containing Ni^2+^, Mn^2+^, and Co^2+^ ions, respectively, compared to 7.7 μg for the pure LP50. The cell containing the mixed TM ions (electrolyte 5) displayed the highest current during discharge, which corresponded to the highest mass of SEI accumulated, 45.2 μg at 0.5 V, indicating a behavior that is to some degree the accumulation of those observed for the individual TM ion electrolytes, again consistent with that observed by AFM (Figure 2).

The behavior observed in electrolyte 3, which contained Mn^2+^, showed somewhat different behavior from the other samples at lower voltages, with the accumulated mass appearing to plateau below ∼0.75 V. However, the increasing current profile below this potential is somewhat incongruous with this finding. Instead, this mass behavior may be due to the accumulation of a thick viscous, swollen, porous, or soft SEI, inducing viscoelastic losses at the crystal that alter its resonance properties and inducing error or nonapplicability of measurement, or the establishment of an interphasial dissolution/deposition equilibrium at the electrolyte and electrode surface. This is possible, as Mn^2+^ induced SEI layers have been demonstrated to be more prone to dissolution.^44−46^ However, AFM data in Figure 2c and Figure S6 suggest that this mass plateau is in fact likely caused by a mix of the two causes, as both a thick, inconsistent, and soft SEI can be observed, but also the layer formed is observed to be somewhat unstable on the surface, especially when the cell is charged back toward 3.0 V.

The MPE (mass per mole of electron transferred) values at different discharge stages can be calculated from the slope of the mass–charge curves using the Sauerbrey equation (see Methods), which can be used to link any reduction in the crystal frequency to a change in the areal mass. However, here it should be noted that the accuracy of his measurement is reduced when nonideal films are deposited; an ideal film is as rigid and homogeneous (same density and low porosity), flat (low roughness), and irreversible (e.g., undissolvable), while not exceeding 2% of the crystal mass.^47^ Nonetheless, while the forming SEI layer was certainly nonrigid and viscoelastic, the MPE values can still be used to give an indication of the composition of the SEI layer during formation.^43^Table S3 shows theoretical MPE values for a range of compounds commonly known to result from LIB electrolyte breakdown at the anode interface.^17,41,43^

The mass–charge curves derived from the EQCM measurements in the five electrolytes between 3.0 and 0.5 V are presented in Figure 4c, and corresponding MPE values at different stages are provided in Table 1. The formation of an SEI on carbon electrodes is generally accepted to follow three steps:^48^

(i) Between 1.5 and 1.0 V, the LiPF_6_ salt reacts with trace amounts of water, and LiF (MPE = 26 g mol^–1^) is generated.
  LiPF_6_ + H_2_O → 2HF + LiF(s) + POF_3_(g)
  HF + Li^+^ + e^–^ → 0.5H_2_(g) + LiF(s)
(ii) Between 1.0 and 0.6 V, EC and EMC solvents are reduced, leading to the deposition of organic SEI species such as (C_2_H_4_OCO_2_Li)_2_ (MPE = 57 g mol^–1^) and LEDC (MPE = 80.9 g mol^–1^).
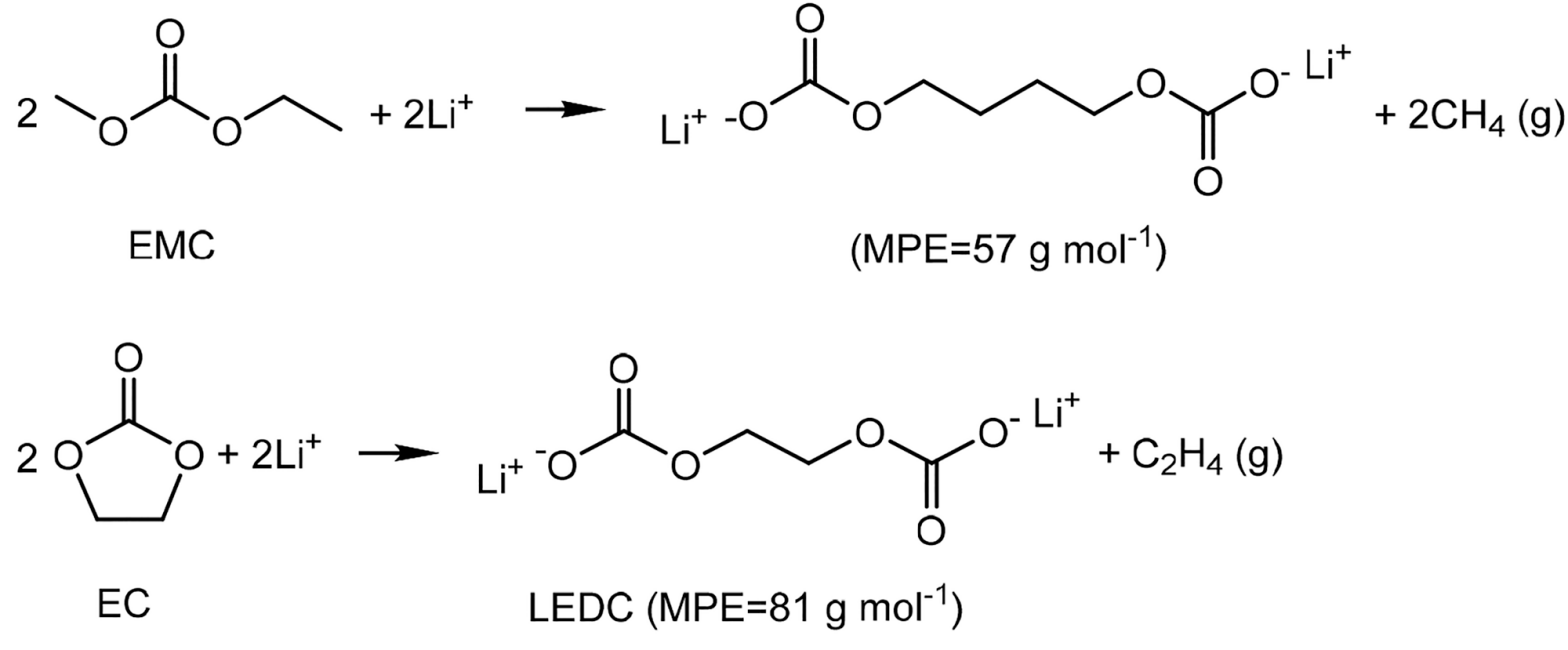
(iii) Hydrolysis of EC by RO^–^ species then leads to the generation of lower-molecular-weight ethylene glycol and ethylene glycol carbonate derivatives (PEG, MPE > 100 g mol^–1^).
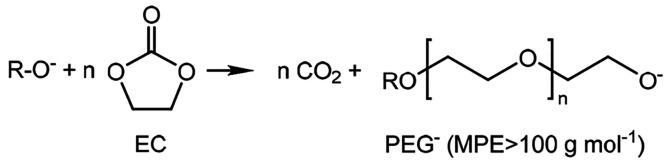

**Table 1:** MPE Values (g mol^–1^) at Different Discharge Stages As Obtained from the Slope of Mass–Charge Curves

	voltage (V)			
	3.0–1.5	1.5–1.0	1.0–0.75	0.75–0.5
(1) LP50	3.8	25.9	47.5	44.9
(2) Ni 800 ppm	38.5	63.7	67.6	90.6
(3) Mn 100 ppm	56.1	94.5	46.4	14.4
(4) Co 100 ppm	94.4	76.4	84.4	52.4
(5) NiMnCo 8/1/1	66.8	108.3	85.3	92.7

In electrolyte 1, containing pure LP50, the MPE values obtained were 25.9, 47.5, and 44.9 g mol^–1^, in the voltage ranges of 1.5–1.0, 1.0–0.75, and 0.75–0.5 V, respectively. This can be used to tentatively suggest that the measured SEI composition shifts from low MPE inorganic species such as LiF (25.9 g mol^–1^) and Li_2_CO_3_ (37 g mol^–1^) to higher MPE species including (C_2_H_4_OCO_2_Li)_2_ (57 g mol^–1^) or polymers such as LEDC (80.9 g mol^–1^), consistent with previous observations in “standard” LIB electrolytes.^38,49^

Significant differences can be observed in the mass accumulation behavior of the electrodes assessed in electrolytes 2 to 5, all showing earlier and higher MPE gains due to the addition of metal ions, consistent with the AFM data (Figure 2). Higher MPE values of 63.7 to 108.3 g mol^–1^ between 3.0 and 1.0 V could be indicative of heavier inorganic metal salts such as NiF_2_ (48.4 g mol^–1^) and NiCO_3_ (59.4 g mol^–1^) or increased contributions from organic/macromolecular components such as LEDC (80.9 g mol^–1^), (CH_2_)_4_(OCOOH)_2_ (89.0 g mol^–1^), or PEG (>100 g mol^–1^).^22^ This evidence, along with that from AFM analysis, suggests that the presence of TM ions in the electrolyte drives the generation of TM compounds in place of LiF and Li_2_CO_3_ at higher potentials (>1.0 V), before the majority of SEI formation has begun. These TM compounds subsequently act as catalytic centers to further induce the decomposition of the electrolyte and the generation of additional SEI, leading to excess SEI with undesirable qualities, such as being thick, porous, loose, swollen, and viscous, largely due the presence of additional elastic organic or polymer species.^50^

*Ex situ* XPS was performed on anodes cycled in electrolytes with and without TM ions to further explore the nature of the TM species at the anode and, thus, their role in promoting the subsequent SEI formation (Figure S12). Unfortunately, due to the complex chemistry of the interface, Ni and Co could not be resolved. However, the Mn 2p region showed two peaks at ∼654 and 643 eV with low signal-to-noise ratio, which would usually be indexed to a Mn(+2), but due to the weak signal this could not exclude the presence of Mn(0). These XPS results showcase the nontrivial nature of characterizing SEI through XPS, further highlighting the benefits *operando* EC-AFM/EQCM measurements may offer.

### EIS Analysis

To help uncover the influence of TM ions on anodes with a directly industrially relevant composition, EIS analysis was conducted in coin cells with commercial graphite electrodes. In a typical RC circuit of graphite–lithium half-cells, there are resistances linked to the bulk (mainly attributed to electrolyte so *R*_e_ is only considered in this work), interface layer (*R*_SEI_, only appear when there is an SEI formed), charge transfer process (*R*_ct_), and diffusion (*W*, Warburg impedance). Between the electrode and electrolyte, an electrical double layer also exists that has capacitive characteristics. However, the characteristics are far different from those of an ideal capacitor due to the complicated structure of the SEI layer, such as porosity, roughness, nonuniform distribution, and leakage capacitance. Hence, the nonideal behavior of the capacitor is compensated by a constant phase element (CPE) in the modeling. The impedance of the CPE is determined by the following equation:
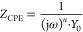
where ω is the angular frequency, *Y*_0_ is the CPE coefficient (pseudocapacitance, unit: μF or mF), *n* is the exponent of the CPE, which is between 0 and 1 (CPE represents a resistor when *n* = 0, a capacitor when *n* = 1, and a Warburg resistance when *n* = 0.5).^51^

**Figure 5:**
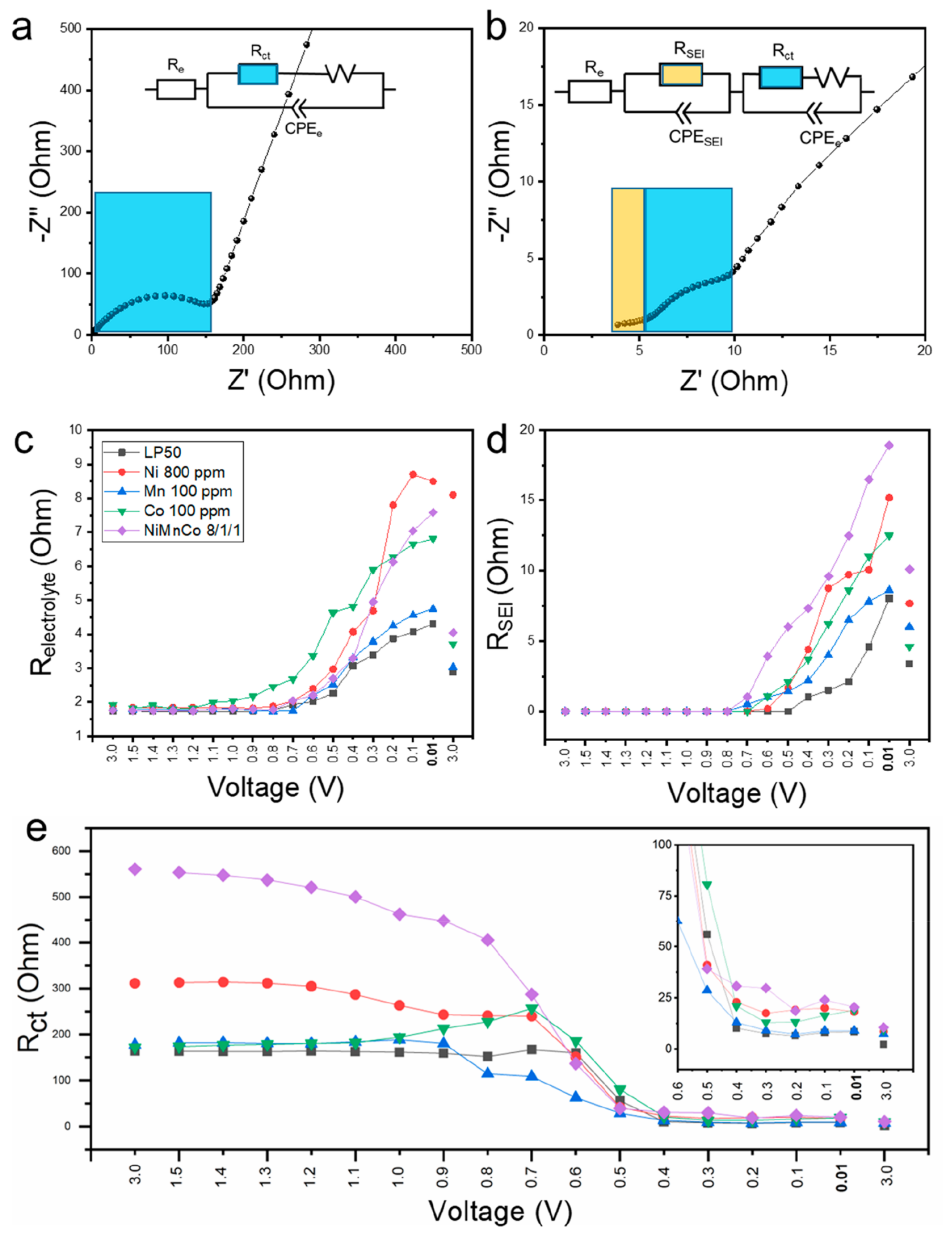
EIS results as measured in coin cells containing composite graphite anodes with the five electrolytes vs Li. Typical Nyquist plot of before (a, OCV) and after (b, 0.01 V) SEI formation in electrolyte 1; insets show the corresponding equivalent circuit. The evolution of the electrolyte resistance (c), SEI layer resistance (d), and resistance of charge transfer (e) during the first discharge/charge for the five samples in (1) LP50, (2) LP50 + 800 ppm of Ni^2+^, (3) LP50 + 100 ppm Mn^2+^, (4) LP50 + 100 ppm of Co^2+^, (5) LP50 Ni^2+^/Mn^2+^/Co^2+^ 800/100/100 ppm. A zoom-in of the response below 0.6 V is shown in (e).

Nyquist plots in Figure 5a and b show typical patterns collected before and after SEI layer formation during the discharge in electrolyte 1. The Nyquist plot before SEI formation (open-circuit voltage (OCV), about 3.0 V, Figure 5a) shows only one semicircle at the high-frequency range, which can be fitted with an equivalent circuit consisting of components including the cell resistance (*R*_e_), a parallel charge transfer resistance (*R*_ct_), a Warburg diffusional resistance (*W*), and a constant phase element of the electrode (CPE_e_). After SEI formation (0.01 V, Figure 5b) the Nyquist plot shows a depressed semicircle at the high-frequency range (yellow) and a bigger semicircle at intermediate frequencies (blue), due to changes in components linked to the SEI, i.e., *R*_SEI_ (resistance of the SEI layer) and CPE_SEI_ (SEI constant phase element) and related changes in charge transfer (*R*_ct_, CPE_e_)_._ These EIS data can be fitted using the equivalent circuit shown in the inset of Figure 5b,^52,53^ and key resistance values measured in the five electrolyte systems (electrolytes 1–5) are compared in Figure 5c–e. The detailed discussion and data of the EIS analysis are provided in Figure S13, Figure S14, and Table S4.

Typically, it has been seen that during the first discharge, *R*_e_ increases due to the degradation of the electrolyte and consumption of Li^+^, *R*_SEI_ emerges and increases while the SEI develops, and *R*_ct_ decreases due to the generation of intercalation/insertion sites in the anode material,^54^ consistent with the data shown here in electrolyte 1. As seen from Figure 5c, all TM ions induce a higher electrolyte resistance than LP50, indicating that electrolytes 2–5 become less conductive than the pure LP50 system (electrolyte 1) during battery operation due to increased degradation of the electrolytes, as seen in EQCM measurements.^54^ Similarly, for all electrolytes, the SEI resistance was consistently higher when TM ions were present (once a non 0 Ω value is achieved upon SEI formation), as shown in Figure 5d, implying that all of the TM ions induce a SEI with lower conductivity. Interestingly, despite the thinner and denser structures observed (Figure 2), Ni^2+^ and Co^2+^ were found to induce SEI resistances higher than those of Mn^2+^, implying that ionic diffusion is easier in the porous Mn^2+^-induced SEI structure. Also consistent with the AFM and EQCM data, the SEI resistance in electrolytes 2–5 increased in magnitude at higher voltages than that in electrolyte 1 due to the presence of TM ions, likely due to their catalytic effect, with the mixed TM ion system (electrolyte 5) showing the earliest SEI formation. For a fixed electrode surface area, *R*_SEI_ and capacitance of the SEI layer (CPE_SEI_) should increase and decrease, respectively, as the SEI layer builds up due to the increasing thickness. However, due to the dynamic nature (changing porous structure and thickness, variable inorganic/organic composition, layer cracking and repairing, etc.) of the SEI layer, the CPE_SEI_ evolution during the first lithiation is complicated.^55^ As seen in Figure S14b, generally, CPE_SEI_ (*Y*_0_) increases as SEI formation begins (0.7 V) and decreases as intercalation occurs (below 0.2 V). Particularly, CPE_SEI_ (*Y*_0_) for electrolytes Mn 100 ppm (3) and NiMnCo 8/1/1 (5) shows higher capacitance than other electrolytes, probably due to the Mn^2+^ induced higher permittivity of the porous organic SEI layer than others.

When the SEI layer was forming during discharge and its resistance (*R*_SEI_) increased, *R*_ct_ could be seen to drop for all cells, due to the development of more/easier to access intercalation/insertion sites in the bulk graphite.^41,56,57^Figure 5e presents the fitted values of *R*_ct_ during discharge in the five electrolytes, which can also be approximated by examining the diameters of the depressed semicircles in the middle-frequency range of the Nyquist plots (Figure S13). In all electrolytes, *R*_ct_ is dropping significantly along with the formation of the SEI layer from 0.7 to 0.5 V, despite an emergence of a small *R*_SEI_. It keeps at a much lower level below 0.5 V due to the favored Li^+^ transportation in the bulk graphite anode at low potential. Electrolytes with Ni^2+^ and Co^2+^ (2, 4, and 5) showed higher *R*_ct_ than that with Mn^2+^ (3) and pure LP50 (1) during the first discharge (as compared at the same voltage), similar to *R*_e_ and *R*_SEI_. Comparatively, *R*_ct_ of electrolyte 3 with Mn^2+^ has slightly higher values than that of electrolyte 1 after SEI formation. In addition, in Figure S14e and f, CPE_ct_ (*Y*_0_) and the Warburg value only increase after the SEI layer is formed and intercalation starts (∼0.2 V), corresponding to the increased difficulty of ionic diffusion at a higher state of charge (>10%).^58^ These results agree well with the previous studies that suggest TM ions can cause some irreversible reactions that prevent interfacial Li^+^ transfer.^24,59^

An interesting consequence of these EIS data is they demonstrate that while the dissolution, migration, and deposition of Mn species is commonly considered to be the main TM-ion-derived deteriorating influence on graphite anode performance,^5,23,60^ Ni^2+^ and Co^2+^ species clearly have a major, possibly equivalent, impact. Therefore, while the above AFM/EQCM results show that Mn ions clearly induce a thicker and softer SEI layer, which is consistent with previous conclusions,^15,16^ the EIS results suggest Ni^2+^ and Co^2+^ cause larger changes in *R*_e_, *R*_SEI_, and *R*_ct_ than Mn^2+^, despite them forming thinner SEI layers. This is important, as high Ni content cathodes, particularly NMCs, are increasingly being seen as the optimal choice for next-generation cells.

To enable overall inter-comparison of the data collected using different methods, it is important to highlight that due to the requirements of the different test methods used, different forms of carbon were tested throughout this work; HOPG was used for EC-AFM, graphite particle coated quartz crystals were used for the EQCM, and composite anodes made from graphite powder were used for coin cell tests. According to previous research, the SEI layer forms differently depending on the structure of the graphite surface, such as particle size, basal-to-edge-plane ratio, pore size, or degree of crystallinity.^17^ However, in this work and in previous studies^25^ it has been shown that, within the same electrolyte, the relative density of edge:basal planes is the primary cause of differences in SEI formation. More basal SEIs will form on HOPG due to its low edge density, which means an edge SEI can be more easily differentiated than in battery-grade graphite particles, which have more edges.^25^ As shown above, there is a greater proportion of inorganic SEI layer on the basal plane, meaning there will be a greater proportion of this on the HOPG. However, as an inorganic SEI is known to have a greater stability, this may indicate that an SEI at graphite particles may be impacted to a greater degree by TM ions. As the EQCM tests used graphite particles with very small particle sizes (∼100 nm), which will possess the smallest basal-to-edge-plane ratio of all the carbons tested and offer a very high surface area, SEI accumulation will be more extreme (as seen by the large SEI accumulations at edges) and more organic (i.e., softer and less stable). This is likely the reason significant mass accumulation was observed. Finally, as the graphite powder used in the composite anodes for coin cell tests had an average particle size of ∼10 μm, the basal-to-edge-plane ratio will be between the former two, thus offering moderate SEI stability, as is common for LIBs. Nonetheless, while these differences are important to note, the data presented show an overall consistency, demonstrating that TM ions significantly impact the type, quantity, and stability of the SEI at graphite anodes, and hence finding strategies to mitigate against this impact will be vital for onward LIB development.

## Conclusions

By correlating fundamental discoveries based on operando EC-AFM and *in situ* EQCM and EIS measurements, the impact of a range of important but problematic cathode-derived TM ions on anode degradation has been revealed. Ni^2+^, Mn^2+^, and Co^2+^ were all found to negatively impact the properties of the SEI formed on graphite anodes, compared to that found in a widely used commercial electrolyte.

EC-AFM showed that Mn^2+^ induces a thick, soft, and unstable SEI layer, all drivers of capacity loss and cell instability of commercial LIBs, while Ni^2+^ and Co^2+^ were observed to increase the accumulation of SEI at the edge plane and basal plane, respectively. All ions were therefore observed to negatively change the structure of the SEI toward one that is known to induce irreversible capacity loss and SEI instability. Force mapping simultaneously showed the SEI formed in the presence of these ions individually was softer, and hence also destabilizing. Speciation via EQCM measurements allowed these degradation modes to be linked to the catalytic behavior of the TM ions, either facilitating enhanced electrolyte decomposition or driving the generation of species that hindered Li^+^ transport. This analysis also demonstrated that when mixed, the impacts of the TM ions combined to accumulate the greatest mass of surface species (including SEI), which AFM analysis showed to be denser, but softer than that formed in electrolytes without TM ions. Via EIS analysis, Ni^2+^ and Co^2+^ were seen to induce particularly significant increases in cell impedance, while in the mixed TM case the SEI resistance increased earlier and to higher levels than all other cells, all of which will also have impacted cell performance by hindering charge transport.

Importantly, while the majority of these findings were based on experiments performed on forming SEI layers, we have also shown through *ex situ* AFM experiments that TM ion concentrations also stimulate increases in the thickness of SEI layers formed without the presence of TM ions, particularly at step edges. This is representative of cathode degradation in real-world cells. Hence, this work reveals that the presence of superfluous TM ions in LIBs drives anode degradation, in particular through SEI destabilization and deactivation. However, this knowledge will enable the design of strategies, including advanced additives, to counter this mode of cell failure in next-generation LIBs.

## Experimental Section

### Materials

Highly oriented pyrolytic graphite (grade ZYB) with a 5 mm × 5 mm area was purchased from Bruker Corp. and connected as the working electrode in an electrochemical cell for EC-AFM. The counter and reference electrodes both consisted of a Ni wire wrapped with a lithium chip (99.9%, MTI Corporation). The LP50 electrolyte contained 1 M LiPF_6_ in EC/EMC (1/1 (v/v)), which was supplied by SoulBrain MI. Nickel(II) and manganese(II) trifluoromethanesulfonate (TFMS) salts (96%) were purchased from Merck Life Science UK Ltd. Cobalt(II) TFMS (98%) was purchased from BLD Pharmatech Ltd. Wrapped crystal EQCM sensors (14 mm) with carbon coatings (AW-R10C10P, sputtered Ti/Au/amorphous carbon, 10/100/100 nm thickness) were purchased from Bio-Logic Science Instruments Ltd. The resonant frequency of the crystal sensors was 10 MHz. Graphite powder (Kaijin AML400) was used to prepare doctor-bladed anodes (13 mg cm^–2^, mixed with PVDF and carbon black with a ratio of 90/5/5) for coin cell (graphite/lithium) CV and EIS tests.

### Methods

The electrolytes were prepared by directly dissolving the TM salts in the commercial LP50 electrolyte (pure LP50 is denoted as electrolyte 1). The concentrations for electrolyte 2 (containing Ni^2+^ from Ni-TFMS), electrolyte 3 (containing Mn^2+^ from Mn-TFMS), and electrolyte 4 (containing Co^2+^ from Co-TFMS) in LP50 were 800, 100, and 100 ppm, respectively (or 0.8, 0.1, and 0.1 mM, compared to Li^+^ at 1,000,000 ppm or 1 M). The influence of mixed TMs was assessed using a nearly stoichiometric ratio of 8:1:1 (Ni^2+^:Mn^2+^:Co^2+^) in LP50 (electrolyte 5).

*Operando* EC-AFM (Bruker Dimension Icon with ScanAsyst) experiments were carried out in an Ar-filled glovebox (Mbraun YKG series) with H_2_O < 0.1 ppm, O_2_ < 0.1 ppm, combined with a CH Instruments electrochemical workstation (model 700E Series Bipotentiostat). PeakForce tapping mode was adopted during imaging with Nu Nano SCOUT 350-silicon probes (Nu Nano Ltd., *k* = 42 N m^–1^, *f*_0_ = 350 kHz). The cell contained a HOPG working electrode (0.12 cm^2^ exposed to electrolyte via a polyimide film covering), Li/Ni counter and reference electrodes, and as-prepared electrolytes (see above). Before experiments, the probe was calibrated on HOPG (modulus = 18 GPa) for a precise measurement of mechanical properties. The images were analyzed using Nanoscope Analysis software (Bruker). The Young’s modulus (alternatively, reduced modulus) was obtained by the Derjaguin–Muller–Toporov (DMT) model. Note that the modulus values of SEI layers on HOPG from different AFM studies vary between tens of MPa and tens of GPa. However, the relative comparison between different samples is reliable because the initial modulus is consistently calibrated to a standard. For EC-AFM experiments, CV was conducted between 3 and 0.01 V at a scan rate of 0.5 mV s^–1^ while the surface morphology, modulus, adhesion, and deformation were recorded concurrently; each image spanned 0.25 V and took 500 s to acquire.

For checking the influence of TM ions on the as-formed SEI layer, a fresh HOPG surface was first exposed to LP50 in an *ex situ* split cell and cycled by CV between 3.0 and 0.01 V at a scan rate of 0.5 mV s^–1^. The cell was then dissembled, gently rinsed with diethyl carbonate to remove excess salt, and dried (in an Ar atmosphere for 10 min) for AFM characterization (in Ar). The same HOPG sample was then reassembled into a split cell, but now with electrolyte 5 (NiMnCo 800/100/100 ppm). After a further same CV, the sample was again dissembled, rinsed, dried, and characterized via AFM.

The EQCM cell was provided by Redox.me AB, Sweden, and used with the carbon-coated crystal sensors described above as working electrodes. Lithium-wrapped Ni wires were used as the reference and counter electrodes. *In situ* EQCM experiments were conducted by correlating LSV tests (Gamry potentiostat, reference 620) with EQCM (Gamry’s eQCM 10M) measurements. LSV scans were carried out from 3.0 to 0.5 V at a scan rate of 0.5 mV s^–1^. The lower voltage was limited to 0.5 V to avoid the alloying of lithium and the underlayer of gold, since most of the SEI layer is established above 0.5 V. The fresh cell was left to equilibrate at open-circuit potential (∼3 V) for 1 h before CV tests. *In situ* EQCM of battery systems enables insights into the SEI mass (Δ*m*) and thickness change during electrochemical reactions to be determined according to Sauerbrey’s equation, where Δ*m* is directly related to any change in the resonant frequency (Δ*f*) of a quartz crystal sensor,
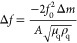
1where *f*_0_ is the resonance frequency of the sensor (here 10 MHz), *A* is the resonator electrode area (here 1.13 cm^2^), and μ_q_ and ρ_q_ are the shear modulus (here 2.947 × 10^11^ g cm^–1^ s^–2^) and density (here 2.648 g cm^–3^) of the quartz crystal, respectively. Assuming the SEI layer is uniform and rigidly attached on the electrode surface, MPE (mass per mole of electron transferred) of deposition matter can be calculated based on the following equation:
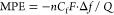
2where *Q* is the charge, *n* is the valence state of reaction ions, *F* is the Faraday constant (96485 C mol^–1^), and *C*_f_ is the mass sensitivity of the sensor. The slope of the *m* versus *Q* plot of the potential step reflects different types of surface film formation processes occurring during the reaction. The sensor surface was characterized by Raman spectroscopy (ThermoScientific DXR Raman microscope with a 532 nm laser) and AFM.

XPS on an uncycled and cycled commercial graphite powder anode (13 mg cm^–2^, mixed with PVDF and carbon black with a ratio of 90/5/5) in electrolyte 1 (LP50) and 5 (NiMnCo 8/1/1) were carried out within split cells, vs Li counter electrode. The cells were cycled by CV at 0.5 mV s^–1^ from the OCV to 1 V vs Li/Li^+^, which is below the metal deposition voltages but above the onset potential of significant SEI formation. The cells were then disassembled, rinsed in DEC (to remove excess LiPF_6_ salts), dried in an Ar atmosphere, and transferred to the XPS in an inert atmosphere for analysis.

For EIS tests, coin cells (CR2032) were assembled with the graphite powder anode and lithium foil, Celgard 2400 separator, and the five electrolytes. The coin cells of different electrolytes (with/without TM ions) were discharged/charged between 3.0 and 0.01 V for 1 cycle, with a constant current density of 0.1 mA cm^–2^ (Gamry potentiostat, reference 620). EIS was taken at open-circuit potential, 1.5, 1.4 to 0.1, 0.01 V, and finally at a charged state of 3.0 V. The potentiostatic EIS test was set from a frequency of 100 kHz to 0.01 Hz, at an AC voltage of 10 mV. Before each of the EIS tests, the cell is stabilized for 1 min at open-circuit potential. The Nyquist plots of the EIS were obtained and fitted with Gamry Echem Analyst.

## Supporting Information Available

Detailed information about the electrochemical tests of coin cells, *in operando*/*ex situ* EC AFM images of the complete discharge/charge experiments, as measured data of height and modulus from the images, properties of QCM crystal sensor, potential SEI components with MPE values, XPS test results and discussion, and Nyquist plots, fitting method, and data of the EIS tests (PDF)
