# Supplementary material for: The Influence of Cathode Degradation Products on the Anode Interface in Lithium-Ion Batteries
Source: ACS Nano. 2024 Mar 20;18(13):9389–402. doi: 10.1021/acsnano.3c10208 (PMC10993644; doi:10.1021/acsnano.3c10208)
Supplement: Supplementary file 1 — nn3c10208_si_001.pdf [file nn3c10208_si_001.pdf]

Supporting Information

The Influence of Cathode Degradation Products on the Anode Interface in Lithium-Ion Batteries

Zhenyu Zhang,[abc] Samia Said,[a] Adam J. Lovett,[ab] Rhodri Jervis,[ab] Paul R. Shearing,[abd] Daniel J.L. Brett,*[ab] Thomas S. Miller*[ab]

[a] Electrochemical Innovation Lab, Department of Chemical Engineering, University College London, Torrington Place, WC1E 7JE, London, UK

[b] The Faraday Institution, Quad One, Becquerel Avenue, Harwell Campus, Didcot, OX11 ORA, UK

[c] Renewable Energy Group, Department of Engineering, Faculty of Environment, Science and Economy, University of Exeter, Penryn Campus, Penryn, TR10 9FE, UK

[d] Department of Engineering Science, University of Oxford, Parks Road, Oxford, OX1 3PJ, UK

*Corresponding author: e-mail: t.miller@ucl.ac.uk, d.brett@ucl.ac.uk

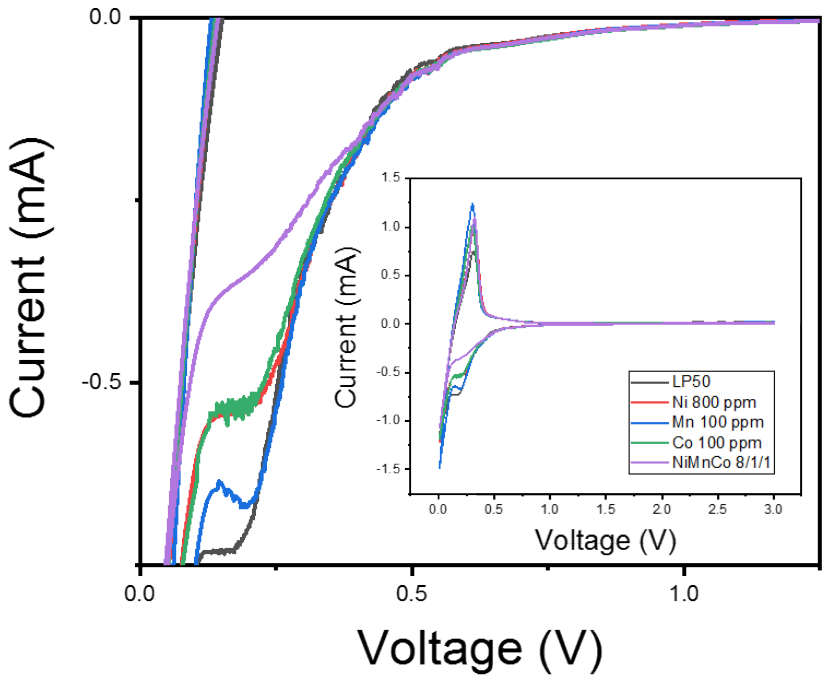

Figure S1. CV curves with scan rate of 0.05 mV s^{-1} of the coin cells of graphite *vs*. Li in the 5 electrolytes. The anode is prepared by mixing graphite powder, carbon black and polyvinylidene fluoride (PVDF) in a ratio of 90/5/5 on Cu foil, with a loading of 13 mg cm^{-2}.

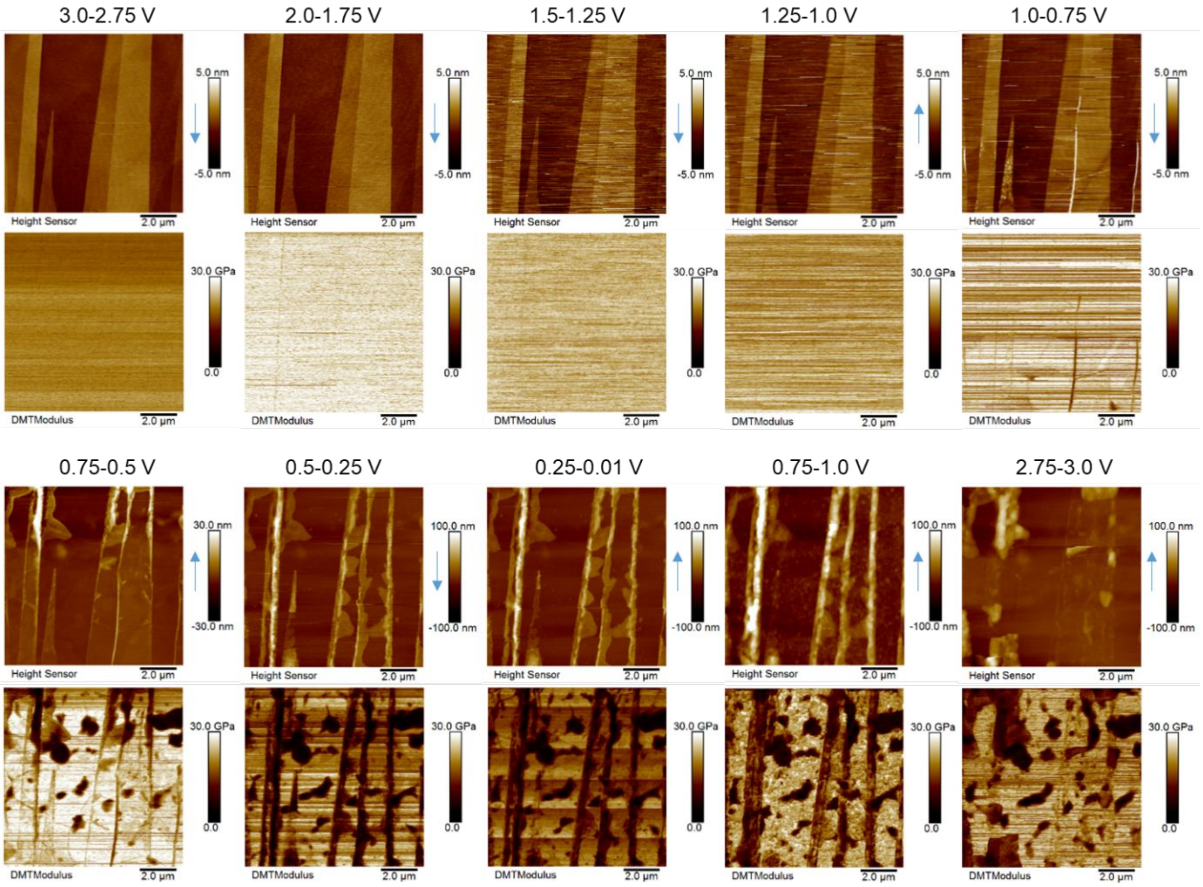

Figure S2. In *operando* EC-AFM images within a 10×10 μm^2 area of height (upper row) and modulus (lower row) mapping during the first discharge and charge of CV scan in the electrolyte (1), LP50.

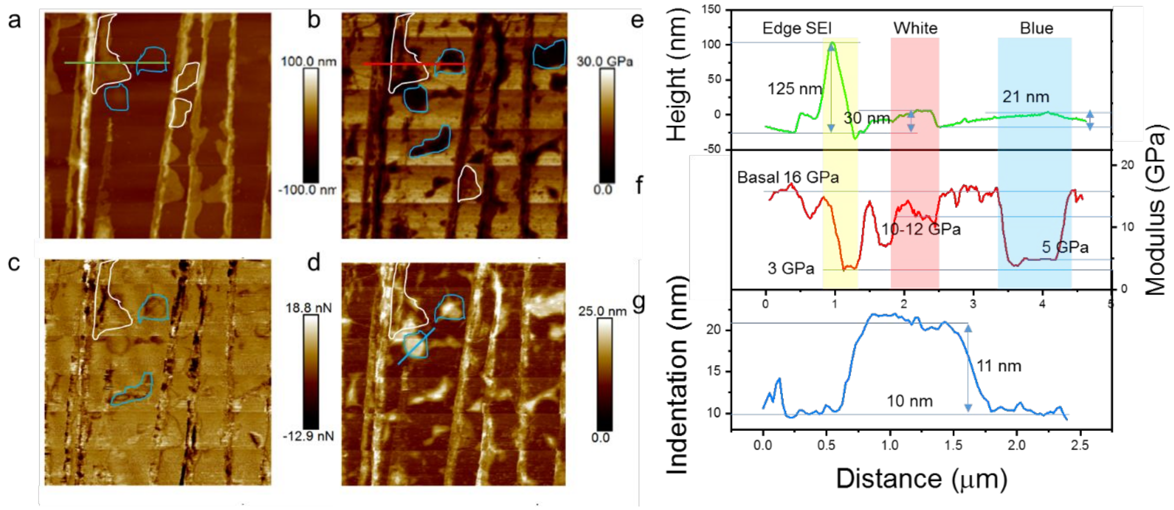

Figure S3. The (a) height, (b) modulus, (c) adhesion, and (d) indentation mapping of 0.25-0.01 V image in **Figure S2** The height profile (green line in (a)), modulus profile (red line in (b)) and indentation depth profile (blue line in (d)) are displayed in (e), (f), and (g) respectively.

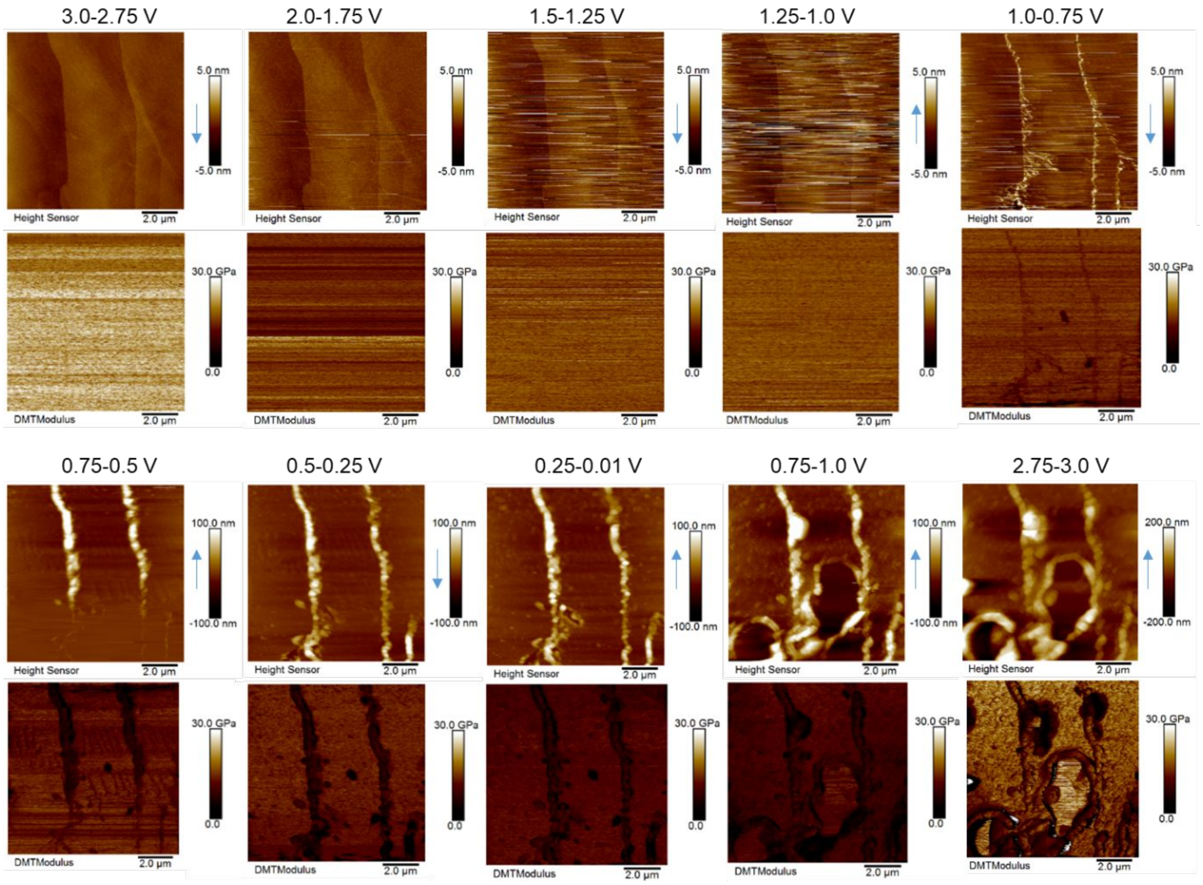

Figure S4. In *operando* EC-AFM images within a 10×10 μm^2 area of height (upper row) and modulus (lower row) mapping during the first discharge and charge of CV scan in electrolyte (2), LP50 with 800 ppm Ni-TFMS.

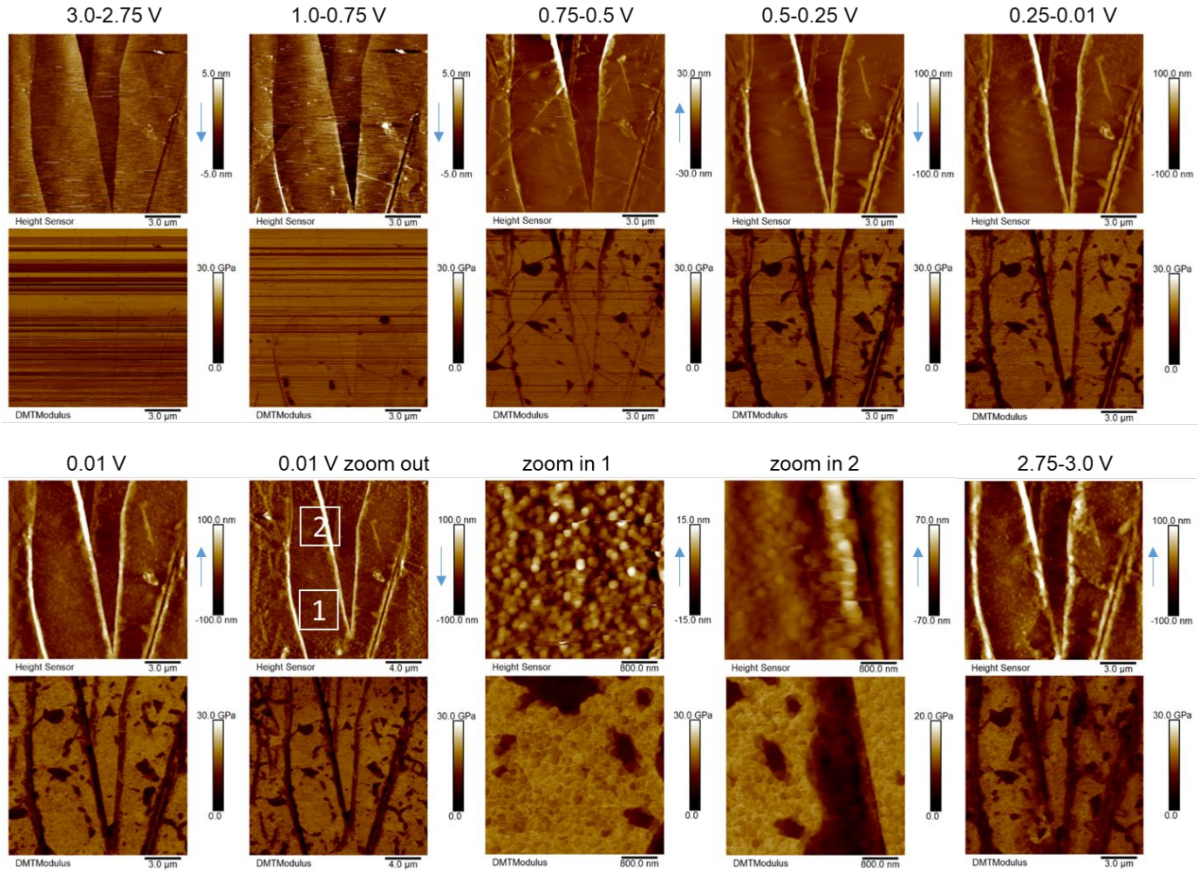

Figure S5. In *operando* EC-AFM images within a 15×15 μm^2 area of height (upper row) and modulus (lower row) mapping during the first discharge and charge of CV scan, in the electrolyte of LP50 with 100 ppm Ni-TFMS for a fair comparison. At 0.01 V, the image is zoomed out to within a 20×20 μm^2 area, and there are zoomed in images of the white box marked area 1 (basal) and area 2 (edge).

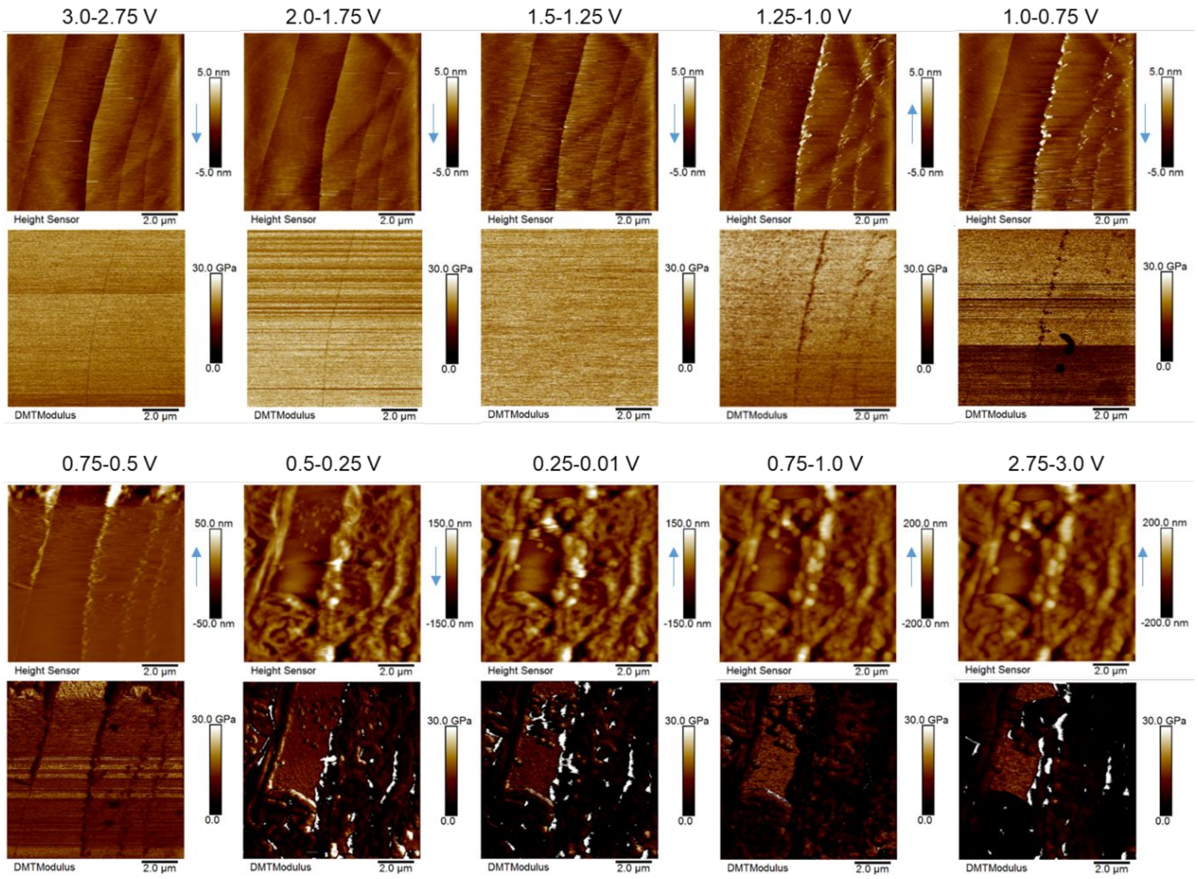

Figure S6. In *operando* EC-AFM images within a 10×10 μm^2 area of height (upper row) and modulus (lower row) mapping during the first discharge and charge of CV scan in the electrolyte (3), LP50 with 100 ppm Mn-TFMS.

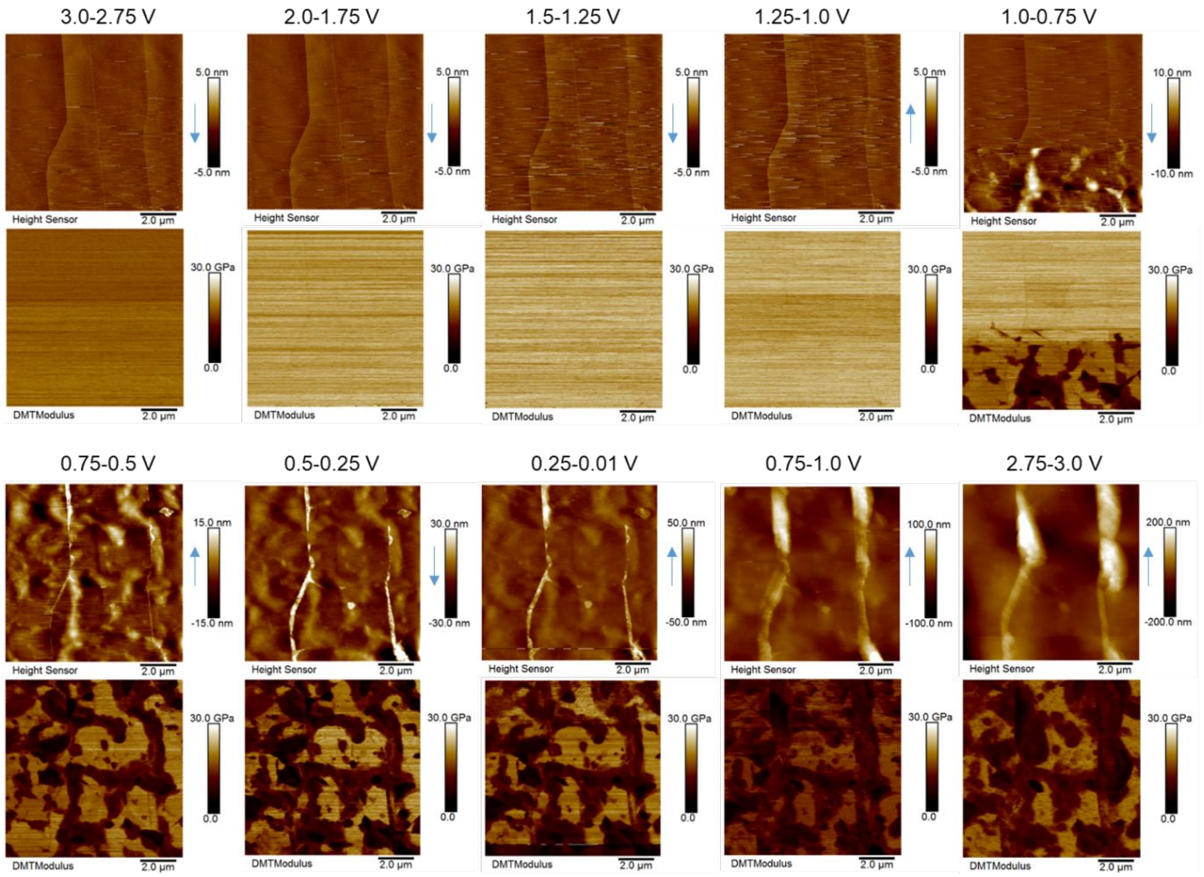

Figure S7. In *operando* EC-AFM images within a 10×10 μm^2 area of height (upper row) and modulus (lower row) mapping during the first discharge and charge of CV scan in electrolyte (4), LP50 with 100 ppm Co-TFMS.

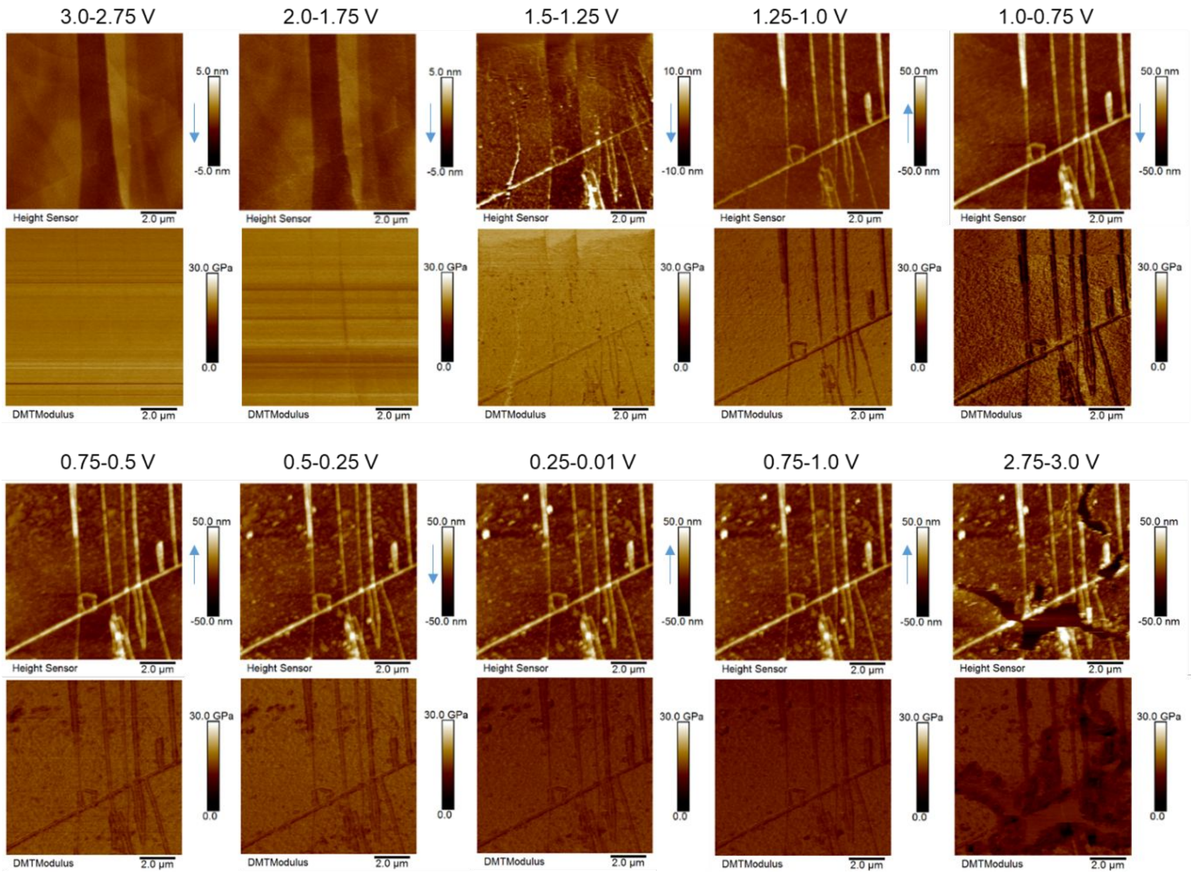

Figure S8. In *operando* EC-AFM images within a 10×10 µm^2 area of height (upper row) and modulus (lower row) mapping during the first discharge and charge of CV scan in electrolyte (5), LP50 with mixed 800 ppm Ni-TFMS, 100 ppm Mn-TFMS, and 100 ppm Co-TFMS.

Edge height (nm)	3.0 V	1.5 V	1.25 V	1.0 V	0.9 V	0.8 V	0.7 V	0.6 V	0.5 V	0.4 V	0.3 V	0.2 V	0.1 V	0.01 V	0.25 V	0.5 V	1.0 V	3.0 V
(1) LP50	3.5	2.6	2.7	2.8	2.4	2.7	11.7	19.7	83.3	106.7	115.0	98.3	95.9	112.8	115.5	102.9	132	92.8
	1.2	2.5	1.7	2.1	2.7	2.5	8.2	11.2	30.3	64.4	62.1	65.9	71.8	64.5	68.8	58.4	78.2	33.0
(2) Ni 800ppm	1	1	1.5	2	4	5	15	121.5	125.2	131.3	104.3	117.3	105.8	108.8	95.0	101.1	104.9	86.8
	1	1	1	1.5	4	5	15	122.2	103.7	94.8	59.9	67.6	111.4	67.4	100.4	67.5	80.7	65.8
(3) Mn 100 ppm	1.4	1.2	1.5	1.5	1.9	2.6	7.3	25.8	57.1	159.3	146	161.8	160	160	141.8	139.7	144.4	140
	2.3	2.8	3	10.2	6.7	11.6	28.2	28.3	95.1	143.7	203.9	181.3	190	200	170	170	150	150
(4) Co 100 ppm	1.2	1.3	1.6	1.6	1.6	1.6	4.6	12	23.2	21.5	30.5	33	45	38.8	45	55	70	150
	0.8	0.7	0.8	1.2	1.3	1.5	5	12.3	16	20.5	30.8	30.5	35.4	30	42	49	56	130
(5) NiMnCo 8/1/1	1.3	2	9.5	48	42.2	47.3	47.4	38.8	42.5	48.3	45.5	40.1	38.8	47.0	42.5	40.3	43.2	41.2
	1.9	2	12.4	32.8	33.2	31.6	38.1	38.4	33.9	34.8	34.9	32.4	34.9	37.0	35.0	36.9	36	37.0

Table S1. The edge height measured from **Figure S2**, **S4**, **S6-S8** at a series of voltage during the EC-AFM. Two edges of each image are selected as marked in **Figure 2**i. There average values are plotted in **Figure 3** for comparison.

Modulus (GPa)	3.0 V	1.5 V	1.25 V	1.0 V	0.9 V	0.8 V	0.7 V	0.6 V	0.5 V	0.4 V	0.3 V	0.2 V	0.1 V	0.01 V	0.25 V	0.5 V	1.0 V	3.0 V
(1) LP50 Edge average	18	23.5	23.8	21.4	23.2	23.2	19	10.2	2.5	2.8	2.6	3.2	3.5	3.1	6.3	7.5	9.5	10.8
Basal average	18	23.5	23.8	21.4	23.2	22.0	23.5	23.5	18	16.5	15.5	15	14.9	15.1	16.5	17	17	17.8
(2) Ni 800ppm Edge average	20.6	15.8	16.9	16.5	13.5	13.2	8.9	4.0	5.5	5.9	5.5	6.6	3.5	4.7	5.2	5.5	7.0	6.0
Basal average	20.6	15.7	16.5	16.3	14.6	14.4	13.0	11.8	12.5	12.5	12.0	11.8	10.9	10.2	9.5	9.0	12.0	17
(3) Mn 100 ppm Edge average	18	19.5	20.2	18.3	16	13	10.5	8.9	7.5	3.4	2.5	1.5	1.3	1.2	1.2	1	1	1
Basal average	18	19.5	20.2	18.3	17	14.25	12.75	11.95	10.55	8.45	7.75	7.25	7.15	7.1	7.15	7.15	7.25	7.5
(4) Co 100 ppm Edge average	18	21	22	22.5	22	17.5	13.5	13.5	7	7.5	6	7	7	5.5	5	6	5.5	6
Basal average	18	21	22	22.5	22	14.5	13.5	12	10	11	11	11	11.5	10	9	10	11	11
(5) NiMnCo 8/1/1 Edge average	18	18	17	12	10.5	10	10.5	11	11.5	11.5	11	11	10	10.5	11.5	11.5	11.5	10
Basal average	18	18	19	16.5	15	15	14	13	13	13	13	12.5	12.5	12.5	12.7	12	12	11.5

Table S2. The corresponding average edge modulus and basal modulus measured from **Figure S2**, **S4**, **S6-S8** at a series of voltage during the EC-AFM. The data are used to plot **Figure 3**.

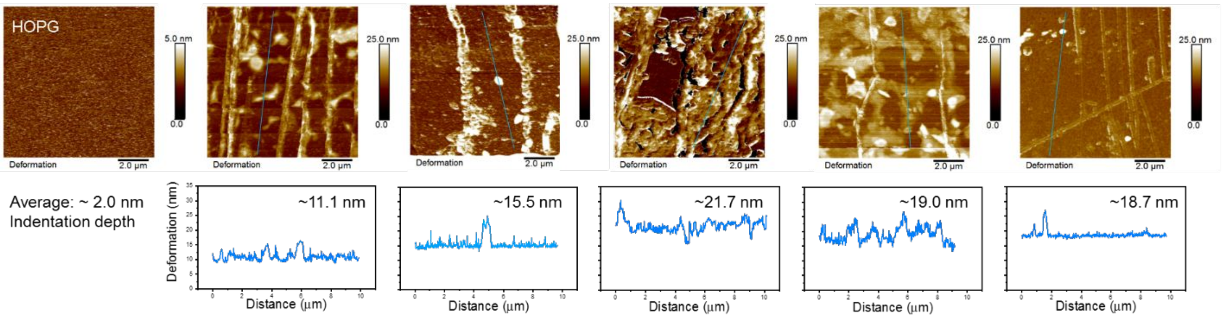

Figure S9. The deformation (indentation depth) image of pristine HOPG, as formed SEI on HOPG in the 5 different electrolytes at discharge state (0.01 V). The deformation profiles along the blue lines in the images at basal areas are presented.

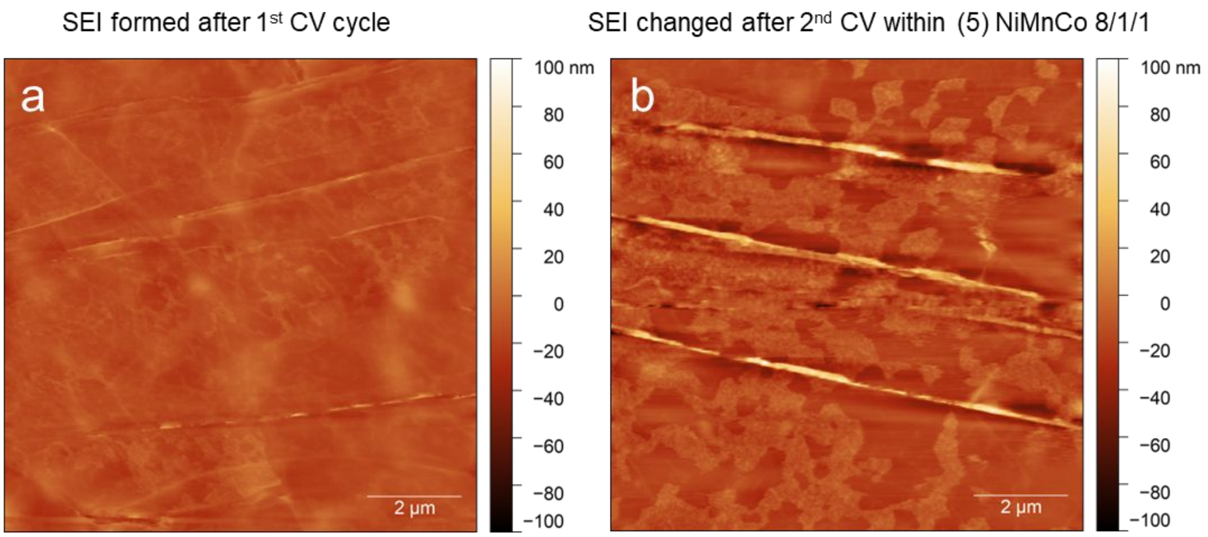

Figure S10. *ex-situ* AFM images of the SEI layer (a) as formed after the 1st CV cycle and (b) as impacted after the 2nd CV cycle conducted in the electrolyte (5) NiMnCo 8/1/1.

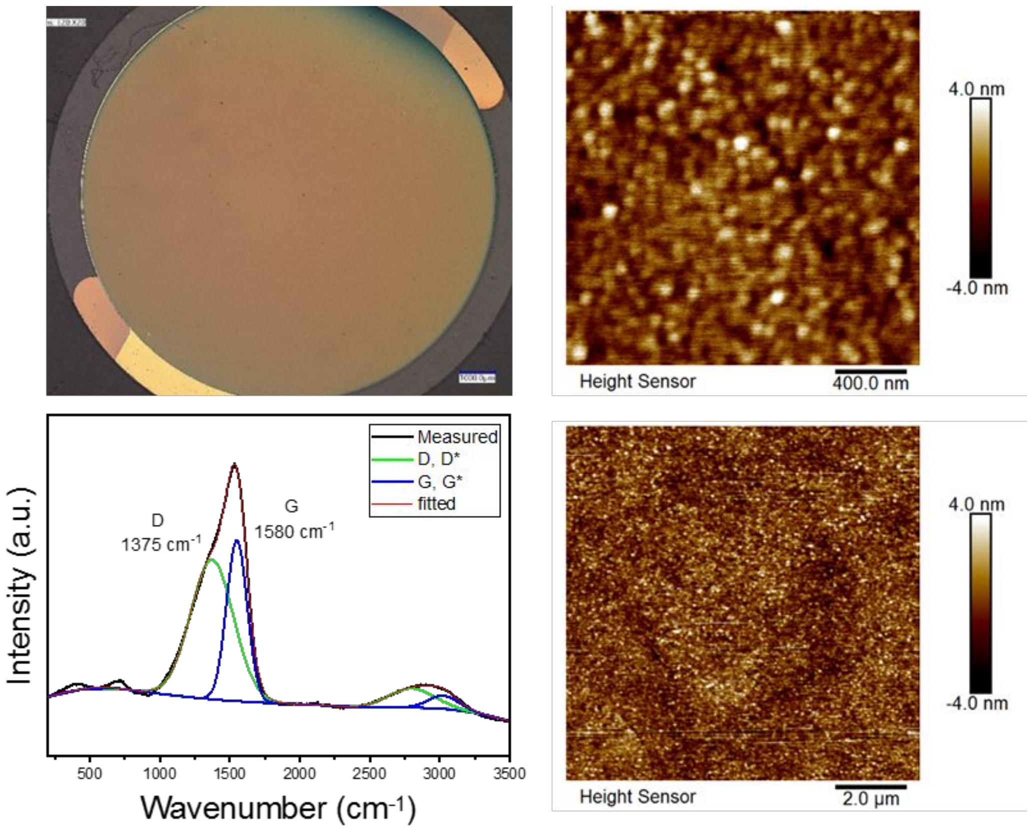

Figure S11. The microscope image, Raman spectroscopy and AFM images of the carbon coated EQCM sensor surface.

Possible species	MPE (g mol^{-1} e^{-})	(1) LP50	(2) Ni 800 ppm	(3) Mn 100 ppm	(4) Co 100 ppm	(5) NiMnCo 8/1/1
LiF	25.9	✓				
Li_2CO_3	37 (74/2)	✓				
Li_2O	15	✓				
$C_2H_4O_2Li_2$	37	✓	✓	✓	✓	✓
$(C_2H_4OCO_2Li)_2$	57	✓	✓	✓	✓	✓
LEDC $(CH_2OCO_2Li)_2$	81	✓	✓	✓	✓	✓
LMC $(LiOCO_2CH_3)$	82	✓	✓	✓	✓	✓
$(CH_2)_4(OCOOH)_2$	89	✓	✓	✓	✓	✓
PEG^{-} $RO(CH_4O)_nCH_4O^{-}$	>100	✓				✓
Ni (0)	29.4 (58.7/2)					
NiF_2	48.4 (96.7/2)		✓			✓
$NiCO_3$	59.4 (118.7/2)		✓			✓
Mn (0)	27.5 (54.9/2)					
MnF_2	46.1 (92.9/2)			✓		✓
$MnCO_3$	57.5 (114.9/2)			✓		✓
Co (0)	29.5 (58.9/2)					
CoF_2	48.5 (96.9/2)				✓	✓
$CoCO_3$	59.1 (118.9/2)				✓	✓

Table S3. presents some of the possible productions decomposed from electrolyte components and their MPE values as a reference for the speculation of SEI species.

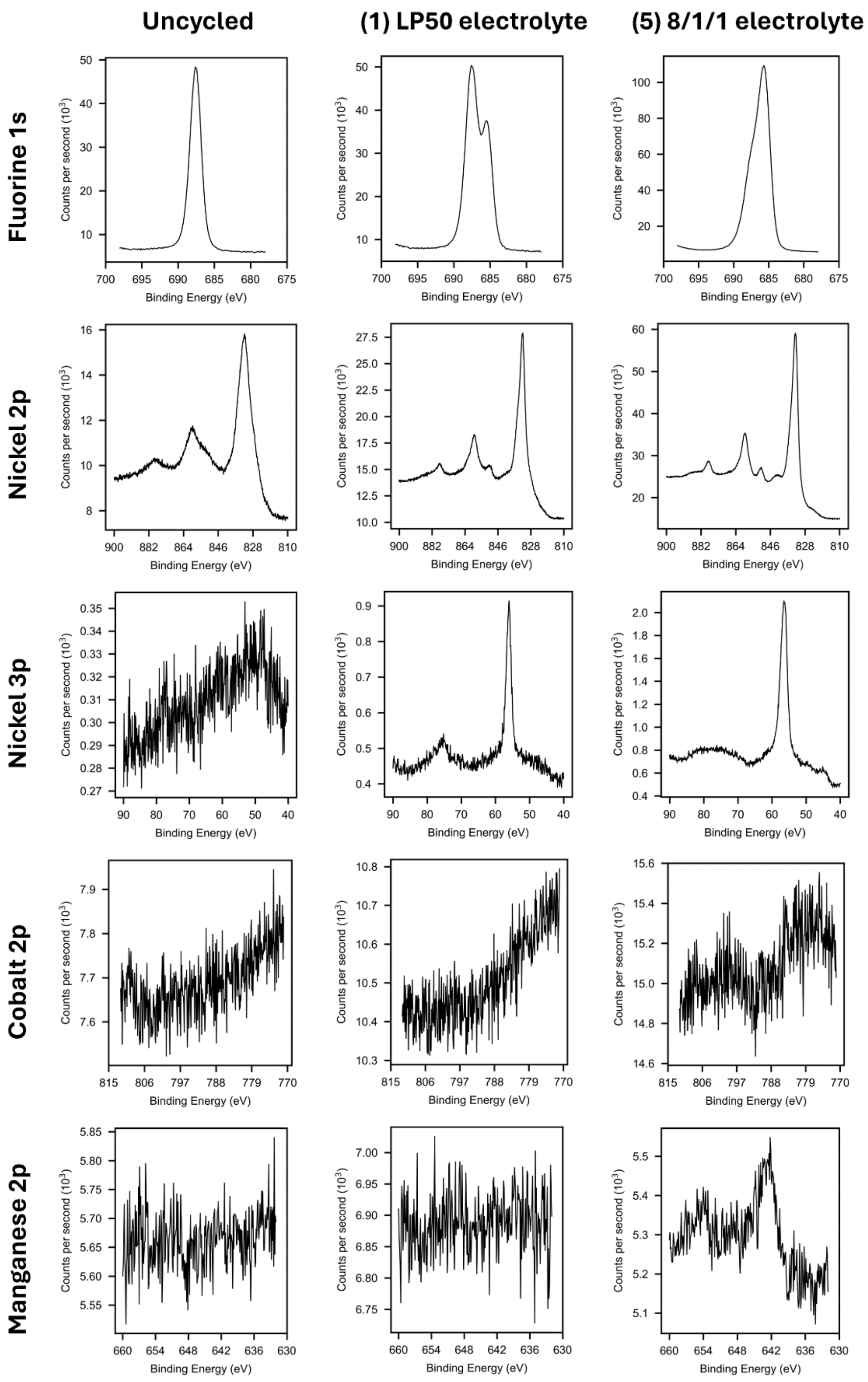

Figure S12. The F 1s, Co 2p, Ni 2p, Ni 3p and Mn 2p regions of XPS spectra obtained from an uncycled graphite anode, a graphite anode cycled in electrolyte (1) LP50 and a graphite anode cycled in electrolyte (5) NiMnCo 8/1/1.

XPS was performed on an uncycled and cycled commercial graphite anode on copper current collector in electrolyte (1) LP50 and (5) NiMnCo 8/1/1. Here a CV was performed at 0.5 mV s^{-1} from OCV to 1 V vs Li/Li^+, well below the metal deposition voltages but above the onset of significant SEI formation at < 0.7 V. The cells were then disassembled, rinsed in DEC (to remove excess $LiPF_6$ salts), and dried in Ar atmosphere, and transferred to the XPS in an inert atmosphere for analysis.

Despite the presence of TM ions in the electrolyte, the peaks observed were of low intensity. Nonetheless, some interesting conclusions can be drawn. From F 1s spectra, F can be seen to be present in all samples. The uncycled sample has one peak in the 1s region (688.0 eV), assigned to the fluorine-containing binder (PDVF). In the cycled (1) LP50 and (5) 8/1/1 electrolyte samples, two peaks are present in the 1s region, which can be assigned again to the binder (688.0 eV) and remnant cycling products (such as LiF, 685.0 eV). Importantly this shows that F containing species associated with the electrolyte are present, despite cycling above the SEI formation voltage, which complicates the analysis of TMs (see below). Interestingly, the intensity of the remnant cycling product (e.g., LiF) component is significantly higher in electrolyte (5) (with respect to the binder) suggesting the TM ions may drive higher levels of electrolyte degradation at these higher voltages.

Unfortunately, this significant F contribution in all samples impacted the ability to resolve Ni, as the Nickel 2p region overlaps with the fluorine Auger KLL peaks. The peaks are nominally at 877 eV, 858 eV and 832 eV (from the fluorine containing polymer) corresponding to the 3 major peaks observed. One or two further low intensity peaks were also observed at ~850 and 845 eV in all samples (broader for uncycled sample), again ascribed to F Auger peaks, noting the splitting observed in the F 1s signal. Further, these extra peaks cannot be assigned to Ni metal or Ni species as no Ni $P_{1/2}$ peak was observed at ~ 870 eV – the expected spin orbit coupling intensity would result in an observable peak. Yet, Ni presence cannot be ruled out however, as the Ni peaks may be overlapped by the F peaks and thus masking the presence of Ni on the sample surface.

We also tested the nickel 3p region, where there is no peak present in all 3 samples at ~67 eV (Ni metal 3p). The sharp peak at 56 eV in the (1) LP50 and (5) 8/1/1 electrolyte is assigned to Li 1s, only present in the cycled samples, as expected. The broad peak between 70-90 eV is ascribed to the Cu 3p (75-80 eV) from the copper current collector. Likewise, Ni presence cannot be ruled out due to overlapping peaks.

For Co 2p, no peaks could be observed in any of the three samples, despite the lack of overlapping contributions in this region. This is particularly surprising as for Mn 2p, two broad peaks were observed in the anode cycled in electrolyte (5) NiMnCo 8/1/1 at ~654 eV and 643 eV. Mn(0) peaks nominally appear at $2p_{1/2}$ 650 eV, $2p_{3/2}$ 639 eV, which may indicate the Mn here exists in a +2 state, rather than metal, however given the low signal-to-noise ratio it is not possible to confirm. No peaks were observed in this region for the anode cycled in electrolyte (1) LP50, or for the uncycled anode.

Qualitatively, these data suggest that Mn is certainly present on the anode surface when cycled in electrolyte (5). Cobalt could be present in untraceably low quantities or any Co species or were removed during electrode rinsing (which is vital to remove excess salts), highly likely if Co existed as cobalt salts (e.g. CoF_2). Nickel presence cannot be ruled out due to overlapping peaks. However, the poor signal-to-noise ratio and overlapping peaks (particularly for Ni XPS) complicates assignment for Ni and Co. Further, these XPS results showcase the non-trivial nature of characterising SEI XPS, namely challenges with dilute signals, overlapping peaks, and the real need for specialist electrochemistry/XPS setups. Here we will draw attention to three publications concerning operando XPS,[1,2] plus operando XAS,[3] that highlight that ex-situ chemical analysis is significantly problematic for the characterisation of delicate interphasial species. Importantly, we believe our operando EC-AFM/EQCM measurements

may offer an alternative route towards the quantification of a variety of species and conditions on the SEI structure and topography, which can complement the complex operando spectroscopy being explored.

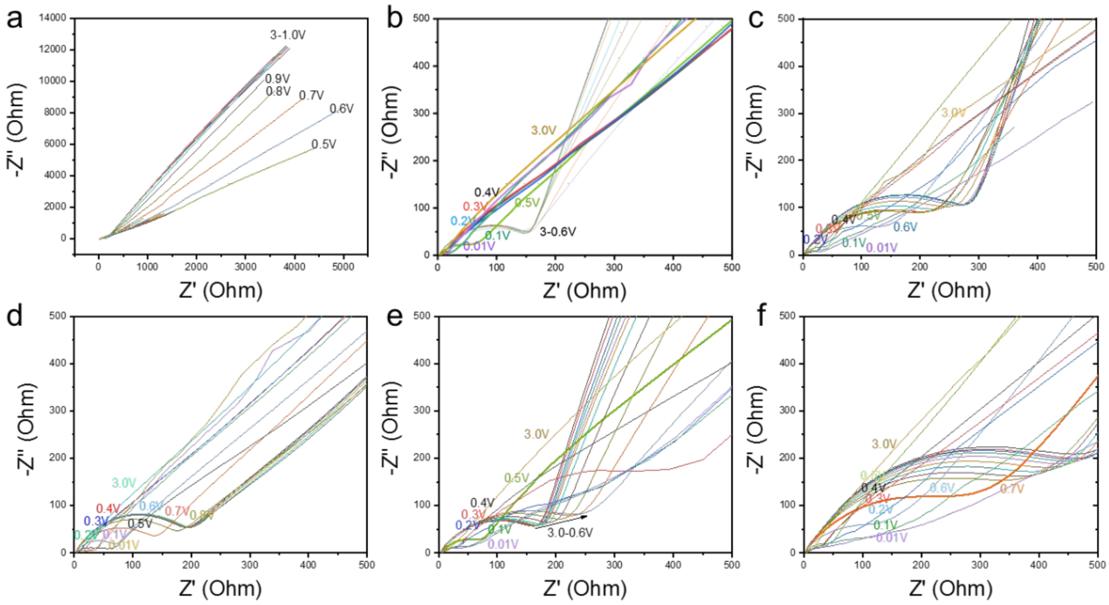

Figure S13. The Nyquist plots of EIS measurements in the discharge/charge process, at different potential and in different electrolytes. (a) and (b) are for LP50, (c) for electrolyte (2), LP50 with 800 ppm Ni-TFMS. (d) for electrolyte (3), LP50 with 100 ppm Mn-TFMS. (e) for electrolyte (4), LP50 with 100 ppm Co-TFMS. (f) for electrolyte (5), LP50 with 800 ppm Ni-TFMS, 100 ppm Mn-TFMS and Co-TFMS.

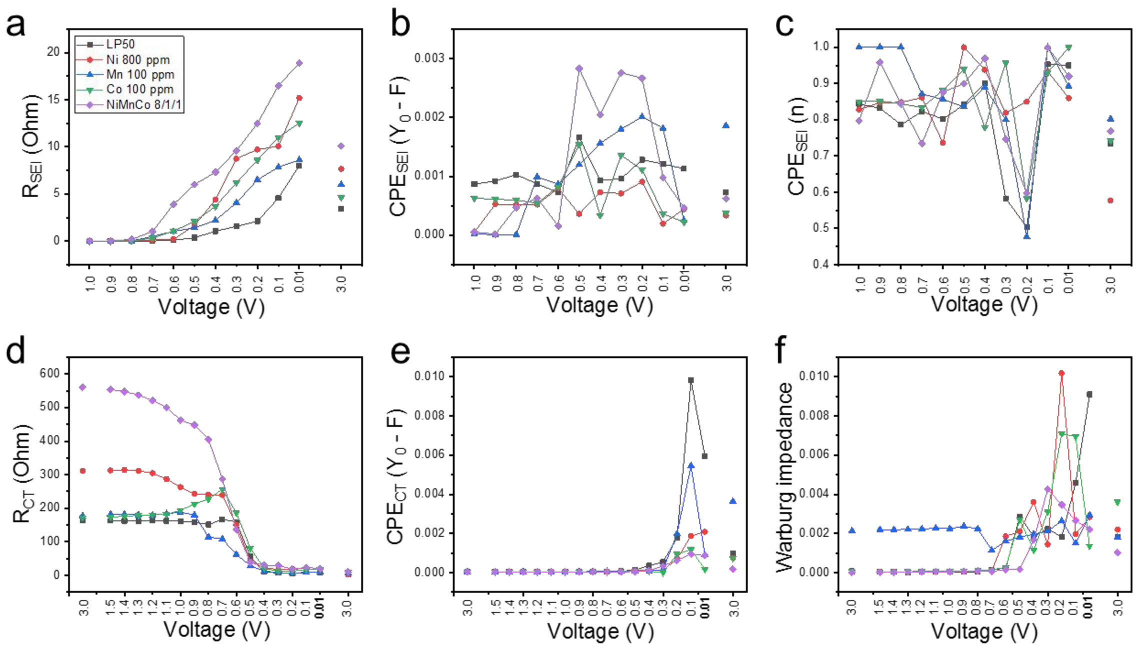

Figure S14. Comparison of the fitting results of different electrolytes. (a) R_{SEI}, (b) CPE_{SEI} (Y_0), (c) CPE_{SEI} (n), (d) R_{CT}, (e) CPE_{CT} (Y_0), and (f) Warburg impedance.

In a typical RC circuit of graphite-lithium half cells, there are resistances linked to the bulk (mainly attributed to electrolyte so R_e is only considered in this work), interface layer (R_{SEI}, only appear when there is SEI formed), charge transfer processed (R_{ct}), and diffusion (W, Warburg impedance). Between the electrode and electrolyte, an electrical double layer also exists that has capacitive characteristics. However, the characteristics are far different from those of an ideal capacitor due to the complicated structure of SEI layer, such as porosity, roughness, nonuniform distribution and leakage capacitance. Hence, the nonideal behaviour of capacitor is compensated by constant phase element (CPE) in the modelling. The impedance of CPE is determined by the equation:

$$Z_{CPE} = \frac{1}{(j\omega)^n \cdot Y_0}$$

Where ω is the angular frequency, Y_0 is the CPE coefficient (pseudo capacitance, unit: μF or mF), n is the exponent of CPE, which is between 0 and 1 (CPE represents a resistor when n=0, a capacitor when n=1, and a Warburg resistance when n=0.5).

Generally, the resistance and capacitance values corresponding to the migration of Li ions through the SEI film can be described as R=ρ l/S and C=ε S/l, where ρ and ε are the resistivity and permittivity constants of SEI film. Since the electrode surface area is fixed, the R and C ($1/Z_{CPE}$) values of SEI film increases and decreases as the building up of SEI layer (increasing of thickness), respectively. However, R and C are not always match this trend, due to the fact that the chemical composition of SEI layer is variable during the formation (thus ρ and ε), so (1) an inorganic layer is first formed above 0.8 V, (2) then a porous organic layer is formed between 0.8 and 0.7 V, (3) an aging process of SEI layer between 0.7 and 0.4 V during which a poor ionic conductivity specie LiF is produced, (4) during the intercalation below 0.3 V, due to the expansion of graphite, SEI layer undergoes cracking and repairing. It is a complicated procedure for the CPE_{SEI} evolution during the first lithiation.

More detailed fitting analysis results are now provided in the following figure and table, including R_e, R_{SEI}, R_{ct}, CPE_{SEI} (Y_0 and n), CPE_{ct} (Y_0 and n) and Warburg resistance. For all the samples, as voltage drops during the first lithiation, Re and R_{SEI} increase (figure 2a 1.0-0.01 V), and R_{ct} also decreases (3.0-0.01 V), as already discussed in the manuscript. Additionally, CPE_{SEI} (Y_0) increases as SEI formation begins (0.7 V) and decreases as intercalation occurs (below 0.2 V). In Figure 2b, CPE_{SEI} (Y_0) for electrolytes (3) Mn 100 ppm and (5) NiMnCo 8/1/1 shows higher capacitance than other electrolytes, probably due to the Mn^{2+} induced higher permittivity of porous organic SEI layer than others. Other than this, CPE_{SEI} (Y_0) and CPE_{SEI} (n) are not showing obvious difference between different electrolytes as the SEI formation is complicated and these values are not stable. In Figure 2e and f, CPE_{ct} (Y_0) and Warburg only increases after SEI layer is formed and intercalation starts (~0.2 V), corresponding to the increased difficulty of ionic diffusion at higher state of charge (>10%). These results are consistent with those in the references provided.

	Voltage (V)	R_b (Ω)	R_{SEI}(Ω)	CPE_{SEI} (Y_0) (S*s^a)	n	R_{CT}(Ω)	CPE_{CT} (Y_0) (S*s^a)	n	W (S*s^(1/2))
(1) LP50	3.0	1.736	-	-	-	163.4	0.046e-3	0.756	0.069e-3
	1.5	1.734	-	-	-	163.1	0.047e-3	0.756	0.048e-3
	1.4	1.726	-	-	-	162.8	0.047e-3	0.755	0.047e-3
	1.3	1.724	-	-	-	162.6	0.047e-3	0.755	0.014e-3
	1.2	1.723	-	-	-	163.7	0.047e-3	0.755	0.042e-3
	1.1	1.722	-	-	-	162.5	0.047e-3	0.755	0.039e-3
	1.0	1.723	0.0009	0.870e-3	0.843	161.2	0.047e-3	0.755	0.034e-3
	0.9	1.725	0.001	0.920e-3	0.832	158.9	0.047e-3	0.756	0.044e-3
	0.8	1.769	0.004	1.035e-3	0.786	151.7	0.048e-3	0.754	0.044e-3
	0.7	1.927	0.016	0.877e-3	0.823	166.8	0.059e-3	0.732	0.146e-3
	0.6	2.015	0.095	0.731e-3	0.802	159.6	0.069e-3	0.709	0.232e-3
	0.5	2.263	0.356	1.661e-3	0.841	55.90	0.142e-3	0.640	2.854e-3
	0.4	3.073	1.052	0.931e-3	0.906	10.16	0.352e-3	0.637	1.853e-3
	0.3	3.388	1.571	0.960e-3	0.583	7.613	0.544e-3	0.924	2.226e-3
	0.2	3.873	2.114	1.283e-3	0.503	6.422	1.780e-3	0.737	1.827e-3
	0.1	4.068	4.568	1.214e-3	0.953	10.05	9.812e-3	1.000	4.585e-3
	0.01	4.299	8.022	1.131e-3	0.950	9.414	5.937e-3	1.000	9.098e-3
	3.0	2.89	3.391	0.735e-3	0.734	2.180	0.994e-3	0.834	1.816e-3
(2) Ni 800 ppm	3.0	1.896	-	-	-	311.1	0.044e-3	0.759	0.042e-3
	1.5	1.843	-	-	-	312.6	0.044e-3	0.760	0.045e-3
	1.4	1.844	-	-	-	313.9	0.044e-3	0.760	0.049e-3
	1.3	1.841	-	-	-	311.5	0.042e-3	0.769	0.032e-3
	1.2	1.846	-	-	-	304.3	0.045e-3	0.759	0.051e-3
	1.1	1.840	-	-	-	286.7	0.046e-3	0.757	0.053e-3
	1.0	1.832	0.015	0.051e-3	0.828	262.9	0.047e-3	0.755	0.051e-3
	0.9	1.824	0.015	0.528e-3	0.847	242.8	0.048e-3	0.752	0.056e-3
	0.8	1.887	0.046	0.521e-3	0.848	240.6	0.052e-3	0.742	0.073e-3
	0.7	2.032	0.125	0.522e-3	0.861	239.5	0.056e-3	0.731	0.104e-3
	0.6	2.392	0.201	0.798e-3	0.736	150.4	0.066e-3	0.737	1.865e-3
	0.5	2.969	1.650	0.363e-3	1.000	40.95	0.114e-3	0.717	2.097e-3
	0.4	4.071	4.400	0.732e-3	0.938	22.85	0.119e-3	0.752	3.606e-3

	0.3	4.688	8.752	0.709e-3	0.819	17.45	0.107e-3	0.804	1.435e-3
	0.2	7.800	9.720	0.908e-3	0.850	19.09	0.706e-3	0.591	10.18e-3
	0.1	8.700	10.07	0.196e-3	0.933	20.01	1.874e-3	1.000	1.976e-3
	0.01	8.500	15.20	0.442e-3	0.860	18.20	2.082e-3	1.000	2.834e-3
	3.0	8.100	7.660	0.337e-3	0.577	2.500	0.751e-3	0.739	2.196e-3
(3) Mn 100 ppm	3.0	1.813	-	-	-	177.3	0.034e-3	0.803	2.130e-3
	1.5	1.777	-	-	-	181.7	0.033e-3	0.803	2.199e-3
	1.4	1.79	-	-	-	181.9	0.033e-3	0.803	2.199e-3
	1.3	1.785	-	-	-	180.4	0.034e-3	0.803	2.207e-3
	1.2	1.764	-	-	-	179.8	0.034e-3	0.803	2.233e-3
	1.1	1.788	-	-	-	183.5	0.034e-3	0.802	2.267e-3
	1.0	1.822	0.012	0.017e-3	1.000	188.3	0.039e-3	0.805	2.245e-3
	0.9	1.747	0.023	0.012e-3	1.000	180.2	0.046e-3	0.800	2.372e-3
	0.8	1.725	0.026	0.012e-3	1.000	114.4	0.055e-3	0.782	2.236e-3
	0.7	1.744	0.501	0.992e-3	0.871	108.4	0.051e-3	0.762	1.141e-3
	0.6	2.250	1.034	0.867e-3	0.856	62.49	0.061e-3	0.758	1.603e-3
	0.5	2.519	1.402	1.198e-3	0.836	28.56	0.078e-3	0.776	1.825e-3
	0.4	3.310	2.205	1.561e-3	0.889	12.94	0.092e-3	0.813	1.966e-3
	0.3	3.781	4.060	1.801e-3	0.800	9.080	0.152e-3	0.841	2.118e-3
	0.2	4.251	6.514	2.011e-3	0.477	7.198	1.980e-3	0.843	2.643e-3
	0.1	4.562	7.840	1.817e-3	1.000	8.904	5.450e-3	0.931	1.505e-3
	0.01	4.75	8.613	0.248e-3	0.893	8.955	0.894e-3	0.753	2.935e-3
	3.0	3.03	6.007	1.860e-3	0.802	7.408	3.632e-3	0.480	1.806e-3
(4) Co 100 ppm	3.0	1.915	-	-	-	171.6	0.038e-3	0.767	0.051e-3
	1.5	1.81	-	-	-	173.3	0.037e-3	0.765	0.046e-3
	1.4	1.904	-	-	-	176.6	0.037e-3	0.765	0.045e-3
	1.3	1.791	-	-	-	178.3	0.037e-3	0.767	0.048e-3
	1.2	1.824	-	-	-	180.1	0.036e-3	0.766	0.056e-3
	1.1	1.987	-	-	-	181.7	0.037e-3	0.763	0.067e-3
	1.0	2.035	0.006	0.627e-3	0.850	193.8	0.038e-3	0.754	0.074e-3
	0.9	2.171	0.004	0.613e-3	0.852	212.5	0.041e-3	0.739	0.088e-3
	0.8	2.452	0.030	0.597e-3	0.844	227.3	0.043e-3	0.727	0.100e-3
	0.7	2.678	0.339	0.544e-3	0.834	256.3	0.042e-3	0.718	0.139e-3
	0.6	3.363	1.065	0.822e-3	0.881	186.0	0.047e-3	0.681	0.272e-3
	0.5	4.635	2.083	1.540e-3	0.940	80.60	0.078e-3	0.692	2.699e-3
	0.4	4.822	3.679	0.337e-3	0.779	20.97	0.074e-3	0.745	1.151e-3
	0.3	5.902	6.199	1.359e-3	0.957	12.89	0.045e-6	0.909	3.103e-3
	0.2	6.267	8.587	1.113e-3	0.583	13.11	0.947e-3	0.718	7.086e-3
	0.1	6.65	11.01	0.364e-3	0.930	16.30	1.173e-3	1.000	6.950e-3
	0.01	6.81	12.53	0.223e-3	1.000	18.99	0.171e-3	0.977	1.358e-3
	3.0	3.713	4.631	0.379e-3	0.742	9.368	0.733e-3	0.645	3.610e-3
(5) Ni Mn Co 8/1/1	3.0	1.761	-	-	-	560.7	0.036e-3	0.760	0.007e-3
	1.5	1.755	-	-	-	553.3	0.036e-3	0.760	0.019e-3
	1.4	1.752	-	-	-	547.4	0.036e-3	0.759	0.022e-3
	1.3	1.750	-	-	-	537.1	0.037e-3	0.759	0.027e-3
	1.2	1.742	-	-	-	520.8	0.037e-3	0.758	0.035e-3

	1.1	1.792	-	-	-	500.1	0.038e-3	0.756	0.052e-3
	1.0	1.802	0.0078	0.055e-3	0.797	462.4	0.038e-3	0.758	0.029e-3
	0.9	1.783	0.011	0.017e-3	0.958	447.5	0.039e-3	0.753	0.087e-3
	0.8	1.775	0.195	0.472e-3	0.845	404.9	0.041e-3	0.751	0.091e-3
	0.7	2.040	1.015	0.630e-3	0.734	287.1	0.048e-3	0.742	0.068e-3
	0.6	2.214	3.900	0.156e-3	0.876	136.4	0.042e-3	0.770	0.149e-3
	0.5	2.698	5.997	2.830e-3	0.900	39.1	0.057e-3	0.783	0.161e-3
	0.4	3.295	7.325	2.047e-3	0.969	30.69	0.109e-3	0.726	1.666e-3
	0.3	4.947	9.601	2.759e-3	0.746	29.7	0.330e-3	1.000	4.253e-3
	0.2	6.127	12.48	2.667e-3	0.597	18.62	0.630e-3	0.515	3.466e-3
	0.1	7.04	16.5	0.980e-3	1.000	23.96	0.952e-3	1.000	2.657e-3
	0.01	7.58	18.9	0.467e-3	0.920	20.4	0.871e-3	1.000	2.221e-3
	3.0	4.04	10.1	0.621e-3	0.769	10.5	0.188e-3	0.606	1.022e-3

Table S4. The fitted data from Gamry Echem analyst software are shown in the following table.

Supporting references

(1) A. Benayad, J. E. Morales-Ugarte, C.C. Santini, R. Bouchet, Operando XPS: a novel approach for probing the lithium/electrolyte interphase dynamic evolution, J. Phys. Chem. A 2021, 125, (4), 1069-1081.

(2) Kevin N. Wood, K. Xerxes Steirer, Simon E. Hafner, Chunmei Ban, Shriram Santhanagopalan, Se-Hee Lee, Glenn Teeter, Operando X-ray photoelectron spectroscopy of solid electrolyte interphase formation and evolution in Li_2S-P_2S_5 solid-state electrolytes, Nat. Commun. 2018, 9, 2490.

(3) Jack E. N. Swallow, Michael W. Fraser, Nis-Julian H. Kneusels, Jodie F. Charlton, Christopher G. Sole, Conor M. E. Phelan, Erik Bjorklund, Peter Bencok, Carlos Escudero, Virginia Perez-Dieste, Clare P. Grey, Rebecca J. Nicholls, Robert S. Weatherup, Revealing solid electrolyte interphase formation through interface-sensitive operando X-ray absorption spectroscopy, Nat. Commun. 2022, 13, 6070.
